# Switching the Conformation of 3,2′:6′,3″-tpy Domains in 4′-(4-*n*-Alkyloxyphenyl)-3,2′:6′,3″-Terpyridines

**DOI:** 10.3390/molecules25143162

**Published:** 2020-07-10

**Authors:** Dalila Rocco, Alessandro Prescimone, Edwin C. Constable, Catherine E. Housecroft

**Affiliations:** Department of Chemistry, University of Basel, BPR 1096, Mattenstrasse 24a, CH-4058 Basel, Switzerland; dalila.rocco@unibas.ch (D.R.); alessandro.prescimone@unibas.ch (A.P.); edwin.constable@unibas.ch (E.C.C.)

**Keywords:** 3,2′:6′,3″-terpyridine, crystal structure, conformation, intermolecular interactions

## Abstract

The preparation and characterization of 4′-(4-*n*-octyloxyphenyl)-3,2′:6′,3″-terpyridine (**8**) and 4′-(4-*n*-nonyloxyphenyl)-3,2′:6′,3″-terpyridine (**9**) are reported. The single crystal structures of 4′-(4-*n*-hexyloxyphenyl)-3,2′:6′,3″-terpyridine (**6**), 4′-(4-*n*-heptyloxyphenyl)-3,2′:6′,3″-terpyridine (**7**), and compounds **8** and **9** have been determined. The conformation of the 3,2′:6′,3″-tpy unit is *trans*,*trans* in **6** and **7**, but switches to *cis*,*trans* in **8** and **9**. This is associated with significant changes in the packing interactions with a more dominant role for van der Waals interactions between adjacent *n*-alkyloxy chains and C–H_methylene_... π interactions in **8** and **9**. The solid-state structures of **6** and **7** with the *n*-hexyloxy and *n*-heptyloxy chains feature interwoven sheets of supramolecular assemblies of molecules, with pairs of *n*-alkyloxy chains threaded through cavities in an adjacent sheet.

## 1. Introduction

**Scheme 1:**
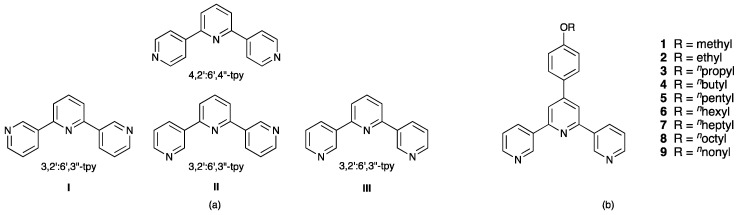
(**a**) Structures of 4,2′:6′,4″-tpy and 3,2′:6′,3″-tpy, and limiting planar conformational variation in 3,2′:6′,3″-tpy. (**b**) Structures of ligands **1**–**9**.

The coordination chemistry of 2,2′:6′,2″-terpyridine (tpy) is well established, and is dominated by tpy acting as a bis-chelating ligand [1,2,3,4,5]. The terpyridine isomers 3,2′:6′,3″-tpy and 4,2′:6′,4″-tpy (Scheme 1a) are growing in popularity as building blocks in coordination polymers owing to the vectorial properties of the outer pyridine *N*-donors, and the fact that the central pyridine ring is coordinatively innocent [6,7,8]. Both 3,2′:6′,3″-tpy and 4,2′:6′,4″-tpy are able to undergo rotation about the inter-annular C–C bonds, but only in 3,2′:6′,3″-tpy does this affect the spatial directionalities of the nitrogen lone pairs of the outer pyridine rings (Scheme 1a). Considering the near-planar conformers, conformation **III** (which can be described as *cis*,*cis*) in Scheme 1a is ideally suited to the formation of discrete [2 + 2] metallocycles, although this has only been structurally confirmed in a few cases [9,10]. We have recently been focusing on investigations of the assemblies of coordination polymers and networks incorporating ligands **1**–**6** (shown in Scheme 1b) and revealed how the planar conformation of the 3,2′:6′,3″-tpy (either **I** or **II**, *trans*,*trans* or *cis*,*trans*, respectively, Scheme 1a) in both [Cu_2_(OAc)_4_L]*_n_* 1D-coordination polymers and [Co(NCS)_2_L_2_]*_n_* 2D-coordination networks responds to changes in the length of the *n*-alkyloxy chain [11,12]. A search of the Cambridge Structural Database (CSD v. 5.4.1 [13]) using Conquest v. 2020.1 [14] reveals 95 entries for the 3,2′:6′,3″-tpy motif, both in free ligands, protonated ligands, and metal coordination compounds. Leaving aside those structures in which the 3,2′:6′,3″-tpy unit is disordered (CSD refcodes YOYGEB, YOYGOL [15], DOGFEN [16], HEZSEL [17], and ZOCFAB [18]), the structural data reveal a dominance of conformation **II** for coordinated ligands.

We focus now on the free ligands. The CSD contains the solid-state structures of only nine free and non-protonated 3,2′:6′,3″-tpy ligands. In two structures, the 3,2′:6′,3″-tpy domain is disordered. In the remaining structures, all of conformations **I**, **II** and **III** are observed (Table 1), but in none of these works has the preference for a given conformation of the 3,2′:6′,3″-tpy been discussed. In our own report of the structure of 1-(3,2′:6′,3″-terpyridin-4′-yl)ferrocene (refcode NEFVEC), we noted that close C–H...N contacts are dominant packing interactions [19].

Our observations that in metal coordination assemblies involving ligands **1**–**6**, the 3,2′:6′,3″-tpy conformation is influenced by the length of the 4-*n*-alkyloxy substituent motivated us to look at the structures of free 4′-(4-*n*-alkyloxyphenyl)-3,2′:6′,3″-terpyridines. Here, we report the syntheses and characterizations of compounds **8** and **9** (Scheme 1b) and the crystal structures of compounds **6**, **7**, **8** and **9**, and we discuss the factors that lead to a switch from conformation **I** to **II** as the *n*-alkyloxy chain increases in length.

## 2. Results and Discussion

### 2.1. Synthesis and Characterization of Compounds ***8*** and ***9***

**Scheme 2:**
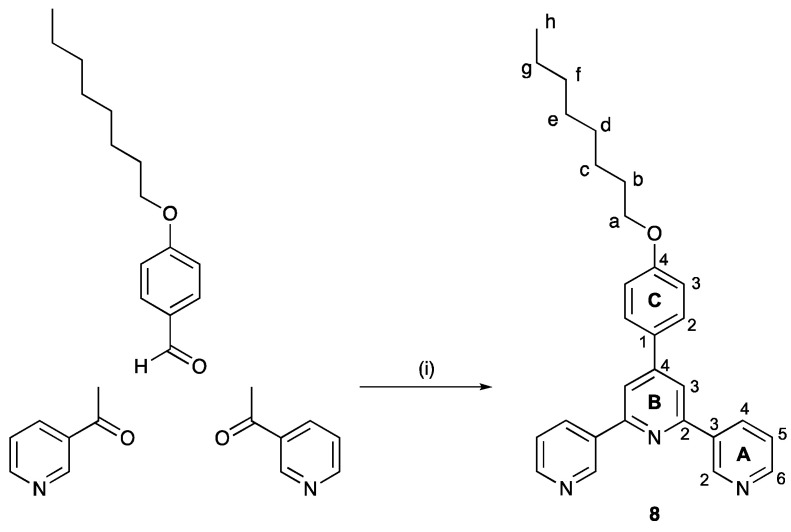
Synthesis of compound **8**; **9** was prepared in a similar manner. Conditions: (i) KOH, EtOH; NH_3_ (aqueous), room temperature. The numbering scheme for NMR assignments is shown; an analogous scheme is used for **9**.

Compounds **6** [25] and **7** [11] were prepared as previously described using the one-pot approach of Wang and Hanan [26], and this methodology was also used to synthesize compounds **8** and **9** (Scheme 2). The electrospray mass spectra of **8** and **9** (Figures S1 and S2 in Supplementary Materials) exhibited base peaks at *m/z* 438.25 and 452.21, respectively, corresponding to the [M + H]^+^ ions. The ^1^H and ^13^C{^1^H} NMR spectra of **8** and **9** are shown in Figures S3–S6 (in the Supplementary Material), and were assigned using COSY, NOESY, HMQC and HMBC techniques. Figures S7–S10 (in the Supplementary Material) display the HMQC and HMBC spectra. The atom numbering used in the experimental section for the NMR assignments is defined in Scheme 2. The ^1^H NMR signals for protons C2 and C3 are readily distinguished using the NOESY crosspeaks between protons B3 and C2. The solid-state IR spectra of **8** and **9** (Figures S11 and S12) are similar, with absorptions at 2917 and 2853 cm^−1^ in the C–H stretching region, and diagnostic strong absorptions in the fingerprint region at 1600, 1518, 1243, 1183, 1022, 704 and 604 cm^−1^ (these values are for **8**).

**Figure 1:**
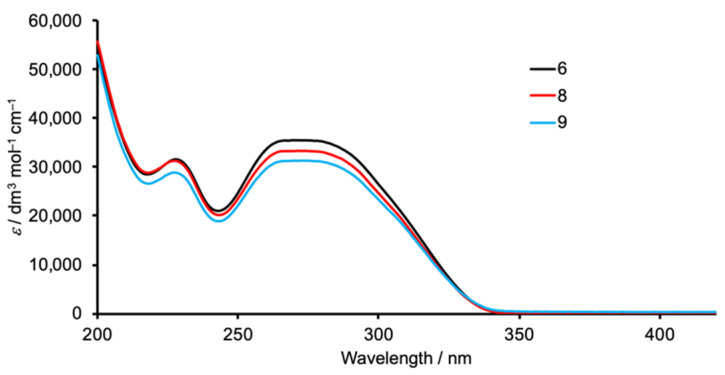
The solution absorption spectrum of compounds **6**, **8** and **9** (MeCN, see Table 2).

The solution absorption spectra of **8** and **9** are shown in Figure 1 and are compared with that of **6**. Although compound **6** has been prepared previously [25], its absorption spectrum has not been reported. The bands arise predominantly from spin-allowed π*←π transitions, and absorption maxima are given in Table 2. The values of λ_max_ compare with 226 and 272 nm in compound **7** [11].

### 2.2. Crystal Structures

**Figure 2:**
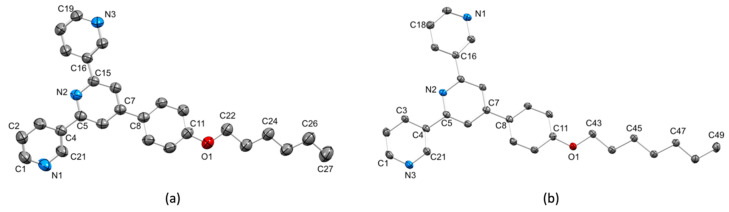
ORTEP representations of one of the two independent molecules of (**a**) **6** and (**b**) **7** (ellipsoids are plotted at a 40% probability level, and H atoms are omitted for clarity). For **6**: C11–O1 = 1.3612(19) Å, C22–O1 = 1.406(2) Å, C11–O1–C22 = 120.10(14)° and for the second independent molecule, the corresponding values are 1.3607(17) Å, 1.4254(18) Å, and 119.30(12)°. For **7**: C11–O1 = 1.3574(19) Å, C43–O1 = 1.4345(18) Å, C11–O1–C43 = 118.51(12)° and for the second independent molecule, the corresponding values are 1.3564(18) Å, 1.4341(18) Å, and 118.15(12)°.

**Figure 3:**
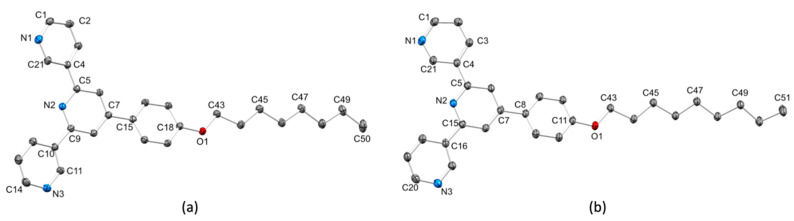
ORTEP representations of one of the two independent molecules of (**a**) **8** and (**b**) **9** (ellipsoids are plotted at a 40% probability level, and H atoms are omitted for clarity). For **8**: C18–O1 = 1.3633(13) Å, C43–O1 = 1.4349(13) Å, C18–O1–C43 = 119.23(8)° and for the second independent molecule, the corresponding values are 1.3630(13) Å, 1.4334(13) Å, and 119.49(8)°. For **9**: C11–O1 = 1.364(3) Å, C43–O1 = 1.437(3) Å, C11–O1–C43 = 118.3(2)° and for the second independent molecule, the corresponding values are 1.365(3) Å, 1.437(3) Å, and 118.22(19)°.

Colorless blocks of **6** were obtained by storing an EtOH solution of the compound for several weeks at 2–5 °C. X-ray quality colorless blocks of **7** were immediately obtained upon recrystallization from EtOH, while colorless blocks of **8** and **9** were obtained by dissolving the compounds in EtOH and storing the solutions for several days at 2–5 °C. Compounds **6**, **7** and **8** crystallize in the monclinic space groups *P*2_1_/*n* (**6**) and *P*2_1_/*c* (**7** and **8**), while **9** crystallizes in the triclinic space group *P* – 1. The asymmetric unit in each structure contains two independent, but structurally similar, molecules and Figure 2 and Figure 3 depict one independent molecule of each compound. Bond lengths and angles are unexceptional. It is worth noting that the C_phenylene_–O bond is shorter than the C_methylene_–O bond (see captions to Figure 2 and Figure 3), consistent with π-conjugation extending from the arene ring to the O atom; the C–O–C bond angles lie in the range 118.18(13)° to 120.10(14)° (captions to Figure 2 and Figure 3) and these values are consistent with *sp*^2^ hybridization at the O atom. The angles between the least squares planes through adjacent pairs of aromatic rings are compiled in Table 3. While the range of values is quite large, there is a general trend for the phenylene/pyridine twist angles to be larger than the pyridine/pyridine twist angles, an observation that is associated with π-stacking interactions between 3,2′:6′,2″-tpy units (see below). The *n-*alkyloxy chain adopts an extended conformation in all the molecules. Again, this is associated with the packing interactions discussed later. Inspection of Figure 2 and Figure 3 reveals that the conformation of the 3,2′:6′,2″-tpy unit changes from **I** (Scheme 1) in compounds **6** and **7**, to **II** (Scheme 1) in **8** and **9**. A detailed look at the molecular packing gives an insight into the reasons for this conformational switch. There is no solvent in the crystal lattice in any of the structures, allowing us to make meaningful comparisons of the crystal packing.

**Figure 4:**
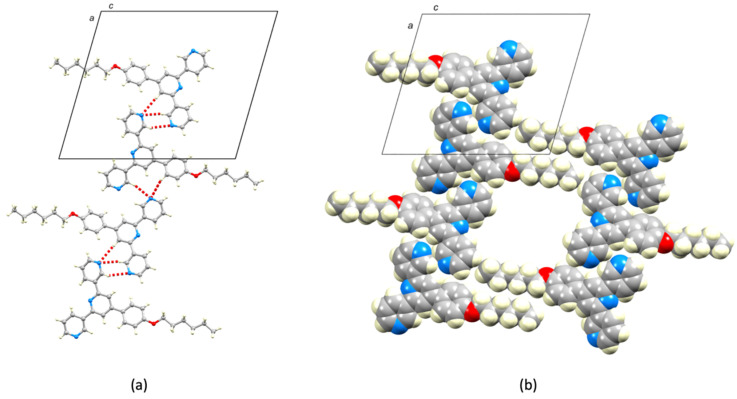
Molecules of **6** pack into 2D-sheets involving CH...N weak hydrogen bonds and van der Waals interactions between *n*-hexyloxy chains. The unit cell is viewed down the *b*-axis. (**a**) C–H...N hydrogen bonds and (**b**) a space-filling representation of part of one supramolecular sheet showing one void between pairs of *n*-hexyloxy chains.

**Figure 5:**
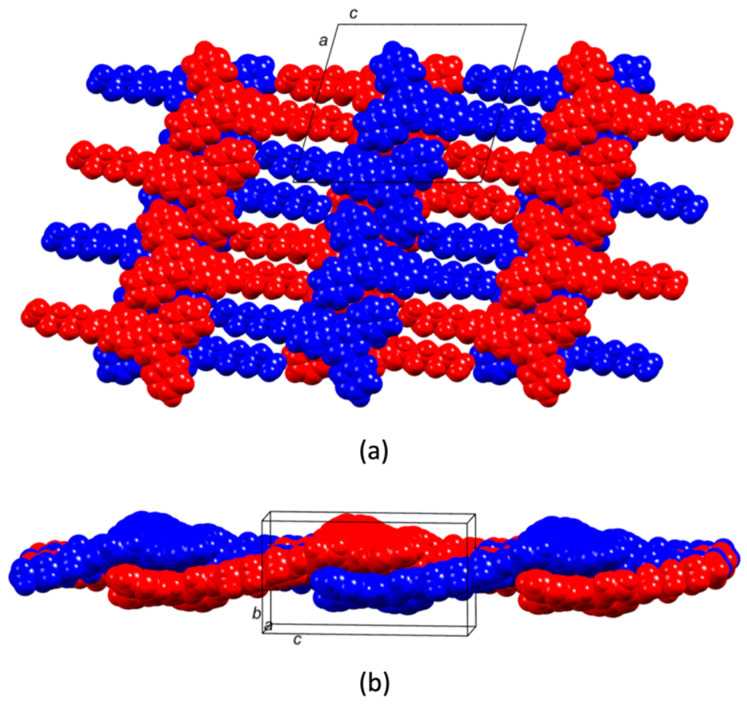
Part of two interwoven supramolecular sheets showing how the voids in one sheet (see Figure 4b) are occupied by *n*-hexyloxy chains from the next sheet. (**a**) Viewed down the *b*-axis, and (**b**) viewed approximately down the *a*-axis.

We start with the packing of molecules of **6**. Pairs of crystallographically independent molecules of **6** interact through C–H...N weak hydrogen bonds (Figure 4a, within the unit cell) with C...N separations of 3.753(2), 3.412(2) and 3.449(2) Å; H...N distances are in the range 2.55–2.83 Å, the H atom being in calculated positions. This motif extends into a ribbon-assembly through bifurcated hydrogen bonds [27] with atoms N3 and N6 acting as bifurcated acceptors with C...N distances of 3.412(2) and 3.753(2) Å for N3, and 3.622(2) and 3.720(2) Å for N6. We note that the N...H interactions are defined by the sum of the Bondi [28] N and H van der Waals radii in the program Mercury [29] with a default value of 1.20 Å for H; a value of 1.10 Å may be more realistic for organic structures [30]. The hydrogen-bonding pattern observed in Figure 4a reflects the fact that both of the independent 3,2′:6′,2″-tpy units exhibit conformation **I**. Figure 4b illustrates centrosymmetric pairing of *n-*hexyloxy chains of adjacent ribbons. The assembly propagates into a non-planar sheet lying approximately in the *ac*-plane, and Figure 4b illustrates that the hydrogen-bonded 2D-network sheet contains voids. Each square-shaped cavity is bordered on two sides by *n*-hexyloxy chains. The voids are occupied by adjacent sheets being woven together (Figure 5a,b), although in a simpler manner than in established biaxial weavings [31]. The interwoven sheets are closely associated through offset face-to-face π-stacking of centrosymmetric pairs of the central pyridine rings of the 3,2′:6′,2″-tpy units (Figure S13 in the Supplementary Materials). For the crystallographically independent molecules of **6**, the π-stacking interactions exhibit inter-plane distances of 3.56 Å and 3.48 Å, and centroid...centroid distances of 3.96 Å and 3.58 Å, respectively. We note that the head-to-tail arrangement of the offset-stacked pyridine rings is optimal in terms of the charge distribution in the pyridine rings [32].

**Figure 6:**
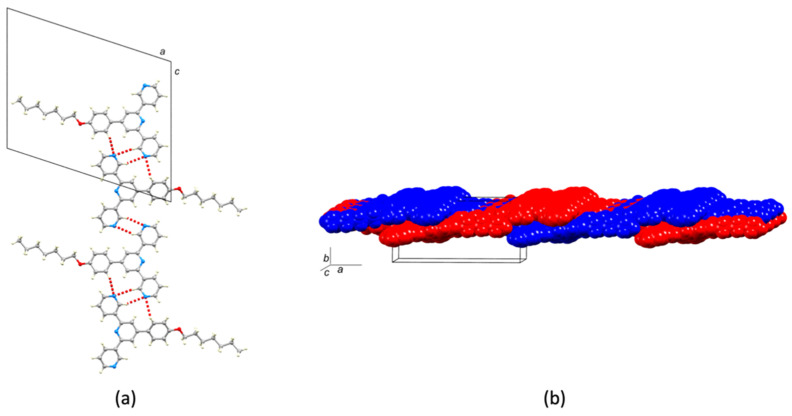
Packing of molecules of **7**. (**a**) Weak C–H...N hydrogen bonds within the supramolecular 2D-sheets and (**b**) interwoven sheets.

Figure 6a shows that molecules of **7** pack into ribbons with a similar C–H...N weak hydrogen bonding pattern as observed in the solid-state structure of **6**, and the same interwoven supramolecular network assembles (Figure 6b). The interwoven network is supported by offset, face-to-face π-stacking of pairs of crystallographically independent molecules of **7** (inter-plane angle = 7.0°, and centroid...centroid distance = 3.58 Å). The similarity between the packing in **6** and **7** negates the need for further discussion of the packing in **7**.

**Figure 7:**
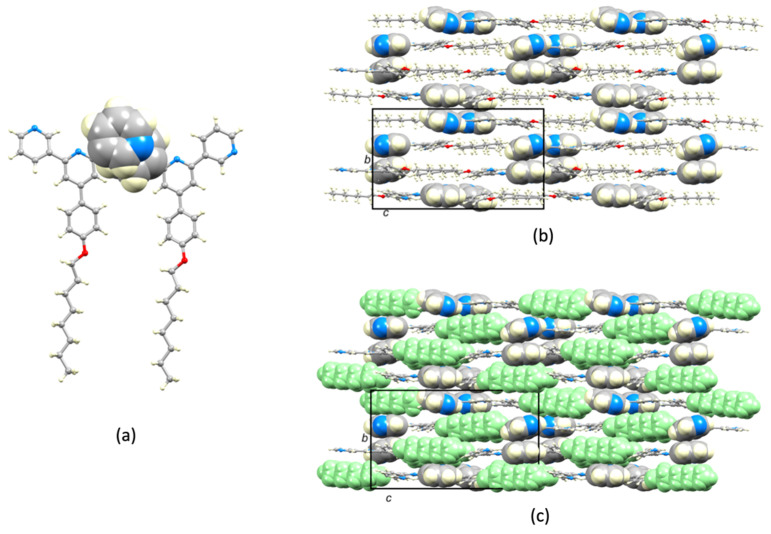
(**a**) Face-to-face π-stacking between pyridine rings (space-filling representation) of crystallographically independent molecules of **8**. (**b**) Molecules of **8** pack into 2D-layers with the *n*-octyloxy chains in extended conformations, and (**c**) the same view of the packing as in (**b**) with the *n*-octyloxy groups in space-filling representation (green).

**Figure 8:**
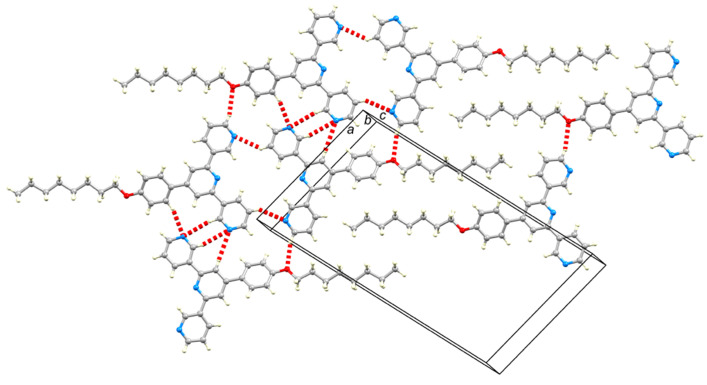
Within each layer of molecules of **8**, van der Waals interactions between *n*-alkyloxy chains, and C–H...N and C–H...O weak hydrogen bonds (red hashed lines) are important packing interactions. See Figure S15 for a space-filling representation.

The packing of the molecules in the lattice undergoes a major change on going from the *n*-hexyloxy and *n*-heptyloxy substituents in **6** and **7** to the longer chains in compounds **8** and **9**, and associated with this is a switch in conformation of the 3,2′:6′,3″-tpy units from **I** to **II** (Scheme 1). In compound **8**, the two crystallographically independent molecules engage in an offset face-to-face π-stacking of the pyridine (py) rings containing N2 and N5 (Figure 7a). The angle between the ring-planes is 14.4° and the centroid...centroid distance is 3.88 Å. This paired motif is a principal packing unit in the lattice. Molecules of **8** pack into 2D-layers with inter-layer py...py π-stacking (Figure 7b,c) being augmented by C–H_methylene_... π interactions [33] as shown in Figure S14 (in the Supplementary Materials). The increase in the length of the *n*-alkyloxy chain leads to a greater role for van der Waals interactions compared to the packing in crystalline **6** and **7**, and this is more important within a 2D-layer (Figure 8 and Figure S15) than between layers. Figure 8 also shows extensive C–H...N and C–H...O weak hydrogen bonding contacts within each 2D-layer, and optimization of these interactions clearly depends upon the conformation of the 3,2′:6′,3″-tpy domain.

**Figure 9:**
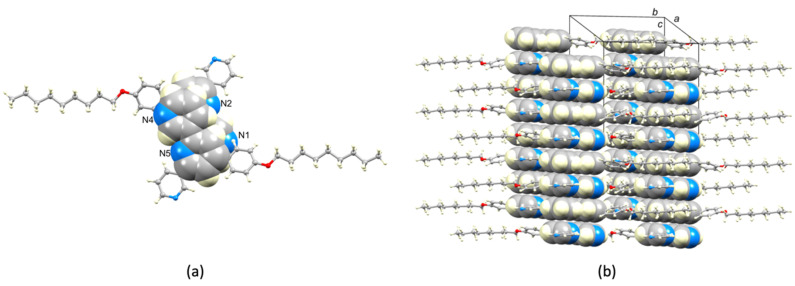
(**a**) Offset face-to-face π-stacking of two crystallographically independent molecules of **9**. (**b**) Packing of molecules of **9** into 2D-layers with the π-stacking shown in (**a**) highlighted in space-filling representation.

**Figure 10:**
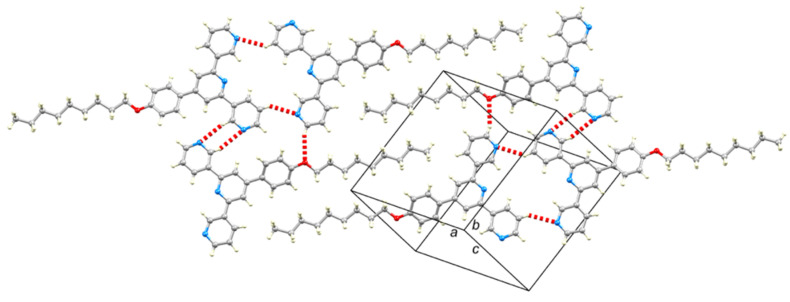
Packing of molecules of **9** within one 2D-sheet involves a combination of C–H...N and C–H...O weak hydrogen bonds (red hashed lines) and van der Waals interactions between *n*-nonyloxy chains. Figure S16 (in the Supplementary Materials) shows this packing in a space-filling representation.

Despite the change in space group on going from **8** (*P*2_1_/*c*) to **9** (*P*–1), there are many similar features in the lattices of the two compounds, indicating that the longer *n*-octyloxy and *n*-nonyloxy chains impart similar influences on the crystal packing. Firstly, the two crystallographically independent molecules of **9** engage in face-to-face π-stacking. In contrast to the motif observed in **8** (Figure 7a), that in **9** involves stacking of the rings containing N1/N5 and those with N2/N4 (Figure 9a). The angles between the planes of the pairs of pyridine rings with N1/N5 and N2/N4 are 8.0° and 1.4°, respectively, and each of the two centroid...centroid distances is 3.82 Å. The fact that each of the two independent 3,2′:6′,2″-tpy domains adopts conformation **II** leads to a favorable arrangement of the stacked pairs of rings in terms of the charge distribution within the heterocycles [32], in keeping with our earlier comments concerning the crystal packing in **6**. As in compound **8**, molecules of **9** are organized in 2D-sheets in which van der Waals interactions between extended *n*-alkyloxy chains play a dominant role (Figure 9b). Figure 10 shows that the packing involves a combination of C–H...N and C–H...O weak hydrogen bonds and van der Waals interactions between *n*-nonyloxy chains.

## 3. Materials and Methods

### 3.1. General

^1^H and ^13^C NMR spectra were recorded on a Bruker Avance iii-500 spectrometer (Bruker BioSpin AG, Fällanden, Switzerland) at 298 K. The ^1^H and ^13^C NMR chemical shifts were referenced with respect to residual solvent peaks (δ TMS = 0). A Shimadzu LCMS-2020 (Shimadzu Schweiz GmbH, 4153 Reinach, Switzerland) instrument was used to record electrospray ionization (ESI) mass spectra. A PerkinElmer UATR Two instrument (Perkin Elmer, 8603 Schwerzenbach, Switzerland) and Shimadzu UV2600 (Shimadzu Schweiz GmbH, 4153 Reinach, Switzerland) or Cary 5000 (Agilent Technologies AG, 4052 Basel, Switzerland) spectrophotometers were used to record FT-infrared (IR) and solution absorption spectra, respectively.

3-Acetylpyridine, 4-*n*-octyloxybenzaldehyde and 4-*n*-nonyloxybenzaldehyde were purchased from Acros Organics (Chemie Brunschwig AG, Basel, Switzerland) and were used as received. Ligands **6** [25] and **7** [11] were prepared as previously reported.

### 3.2. Compound ***8***

4-*n*-Octyloxybenzaldehyde (2.34 g, 2.39 mL, 10.0 mmol) was dissolved in EtOH (50 mL), then 3-acetylpyridine (2.42 g, 2.20 mL, 20.0 mmol) and crushed KOH (1.12 g, 20.0 mmol) were added to the solution. Aqueous NH_3_ (32%, 38.5 mL) was slowly added to the reaction mixture and this was stirred at room temperature overnight. The solid that formed was collected by filtration, washed with EtOH (3 × 10 mL), recrystallized from EtOH and dried *in vacuo*. Compound **8** was isolated as a colorless powder (0.942 g, 2.15 mmol, 21.5%). M.p. = 99 °C. ^1^H NMR (500 MHz, CDCl_3_): δ/ppm = 9.37 (dd, *J* = 2.2, 0.6 Hz, 2H, H^A2^), 8.70 (dd, *J* = 4.8, 1.6 Hz, 2H, H^A6^), 8.50 (dt, *J* = 8.0, 1.8 Hz, 2H, H^A4^), 7.92 (s, 2H, H^B3^), 7.70 (m, 2H, H^C2^), 7.45 (ddd, *J* = 8.0, 4.8, 0.7 Hz, 2H, H^A5^), 7.06 (m, 2H, H^C3^), 4.04 (t, *J* = 6.6 Hz, 2H, H^a^), 1.83 (m, 2H, H^b^), 1.49 (m, 2H, H^c^), 1.41−1.26 (m, 8H, H^d+e+f+g^), 0.90 (m, 3H, H^h^). ^13^C{^1^H} NMR (500 MHz, CDCl_3_): δ/ppm = 160.6 (C^C4^), 155.5 (C^A3^), 150.5 (C^A6^), 150.3 (C^B4^), 148.6 (C^A2^), 135.0 (C^A4^), 134.7 (C^B2^), 130.3 (C^C1^), 128.4 (C^C2^), 123.7 (C^A5^), 117.3 (C^B3^), 115.4 (C^C3^), 68.4 (C^a^), 32.0 (C^d^), 29.5 (C^e/f^), 29.4 (C^e/f^), 29.3 (C^b^), 26.2 (C^c^), 22.8 (C^g^), 14.3 (C^h^). UV-VIS (CH_3_CN, 5 × 10^−5^ mol·dm^−3^) λ/nm 227 (ε/dm^3^·mol^−1^·cm^−1^ 31,292), 271 (33,350). ESI-MS *m/z* 438.25 [M + H]^+^ (calc. 438.25). Found C 79.31, H 7.12, N 9.52; required for C_29_H_31_N_3_O: C 79.60, H 7.14, N 9.60. X-ray quality, colorless block-like single crystals of **8** were obtained upon recrystallization from EtOH and after storing the solution for a few days at 2–5 °C.

### 3.3. Compound ***9***

4-*n*-Nonyloxybenzaldehyde (2.48 g, 2.57 mL, 10.0 mmol) was dissolved in EtOH (50 mL) and 3-acetylpyridine (2.42 g, 2.20 mL, 20.0 mmol) and crushed KOH (1.12 g, 20.0 mmol) were added to the solution. Aqueous NH_3_ (32%, 38.5 mL) was slowly added to the reaction mixture and this was stirred at room temperature overnight. The solid that formed was collected by filtration, washed with EtOH (3 × 10 mL), recrystallized from EtOH and dried *in vacuo*. Compound **9** was isolated as a colorless powder (0.757 g, 1.68 mmol, 16.8%). M.p. = 106 °C. ^1^H NMR (500 MHz, CDCl_3_): δ/ppm = 9.37 (d, *J* = 2.0 Hz, 2H, H^A2^), 8.70 (dd, *J* = 4.7, 1.2 Hz, 2H, H^A6^), 8.50 (m, 2H, H^A4^), 7.92 (s, 2H, H^B3^), 7.71 (m, 2H, H^C2^), 7.46 (m, 2H, H^A5^), 7.06 (m, 2H, H^C3^), 4.04 (t, *J* = 6.6 Hz, 2H, H^a^), 1.83 (m, 2H, H^b^), 1.49 (m, 2H, H^c^), 1.41–1.24 (m, 10H, H^d+e+f+g+h^), 0.89 (m, 3H, H^i^). ^13^C{^1^H} NMR (500 MHz, CDCl_3_): δ/ppm = 160.6 (C^C4^), 155.4 (C^A3^), 150.6 (C^B4^), 150.3 (C^A6^), 148.5 (C^A2^), 135.0 (C^A4^), 134.7 (C^B2^), 130.2 (C^C1^), 128.4 (C^C2^), 123.8 (C^A5^), 117.3 (C^B3^), 115.4 (C^C3^), 68.4 (C^a^), 32.0 (C^b/d/e/f/g/h^), 29.7 (C^b/d/e/f/g/h^), 29.6 (C^b/d/e/f/g/h^), 29.4 (C^b/d/e/f/g/h^), 29.3 (C^b/d/e/f/g/h^), 22.8 (C^b/d/e/f/g/h^), 26.2 (C^c^), 14.3 (C^i^). UV-VIS (CH_3_CN, 2 × 10^−5^ mol·dm^−3^) λ/nm 227 (ε/dm^3^·mol^−1^·cm^−1^ 28,910), 273 (31,350). ESI-MS *m/z* 452.21 [M + H]^+^ (calc. 452.27). Found C 79.70, H 7.22, N 9.38; required for C_30_H_33_N_3_O: C 79.79, H 7.37, N 9.30. X-ray quality, colorless block-like single crystals of **9** were obtained upon recrystallization from EtOH and after storing the solution for several days at 2–5 °C.

### 3.4. Crystallography

Single crystal data were collected on a Bruker APEX-II diffractometer (CuKα radiation) with data reduction, solution and refinement using the programs APEX [34], ShelXT [35], Olex2 [36] and ShelXL v. 2014/7 [37], and or using a STOE StadiVari diffractometer equipped with a Pilatus300K detector and with a Metaljet D2 source (GaKα radiation) and solving the structure using Superflip [38,39] and Olex2 [36]; the model was refined with ShelXL v. 2014/7 [37]. Structure analysis including the ORTEP representations, used CSD Mercury 2020.1 [29].

### 3.5. Compound ***6***

C_27_H_27_N_3_O, *M*_r_ = 409.51, colorless block, monoclinic, space group *P*2_1_/*n*, *a* = 18.8773(3), *b* = 11.3154(2), *c* = 21.6453(3) Å, *β* = 105.985(1)°, *V* = 4444.75(12) Å^3^, *D*_c_ = 1.224 g cm^−3^, *T* = 300 K, *Z* = 8, μ(Ga*Kα*) = 0.378 mm^−1^. Total 34613 reflections, 8962 unique (*R_int_* = 0.0210). Refinement of 7622 reflections (562 parameters) with *I* > 2σ(*I*) converged at final *R*_1_ = 0.0537 (*R*_1_ all data = 0.0606), *wR*_2_ = 0.1586 (*wR*2 all data = 0.1702), gof = 1.055. CCDC 2009745.

### 3.6. Compound ***7***

C_28_H_29_N_3_O, *M*_r_ = 423.54, colorless block, monoclinic, space group *P*2_1_/*c*, *a* = 23.1434(10), *b* = 10.8165(5), *c* = 18.7860(9) Å, *β* = 108.115(3)°, *V* = 4469.6(4) Å^3^, *D*_c_ = 1.259 g cm^−3^, *T* = 130 K, *Z* = 8, μ(Cu*Kα*) = 0.601 mm^−1^. Total 30119 reflections, 8165 unique (*R_int_* = 0.0366). Refinement of 6195 reflections (579 parameters) with *I* > 2σ(*I*) converged at final *R*_1_ = 0.0429 (*R*_1_ all data = 0.0615), *wR*_2_ = 0.1090 (*wR*2 all data = 0.1205), gof = 1.025. CCDC 2009746.

### 3.7. Compound ***8***

C_29_H_31_N_3_O, *M*_r_ = 437.57, colorless block, monoclinic, space group *P*2_1_/*c*, *a* = 13.8447(15), *b* = 14.1626(16), *c* = 24.757(3) Å, *β* = 102.213(4)°, *V* = 4744.4(9) Å^3^, *D*_c_ = 1.225 g cm^−3^, *T* = 130 K, *Z* = 8, μ(Cu*Kα*) = 0.582 mm^−1^. Total 31983 reflections, 8603 unique (*R_int_* = 0.0286). Refinement of 7947 reflections (597 parameters) with *I* > 2σ(*I*) converged at final *R*_1_ = 0.0378 (*R*_1_ all data = 0.0407), *wR*_2_ = 0.1038 (*wR*2 all data = 0.1067), gof = 1.035. CCDC 2009748.

### 3.8. Compound ***9***

C_30_H_33_N_3_O, *M*_r_ = 451.59, colorless block, triclinic, space group *P*–1, *a* = 11.6102(4), *b* = 13.9680(5), *c* = 16.4766(6) Å, *α* = 95.198(3), *β* = 99.538(3), *γ* = 108.195(3)°, *V* = 2474.27(16) Å^3^, *D*_c_ = 1.212 g cm^−3^, *T* = 130 K, *Z* = 4, μ(Ga*Kα*) = 0.368 mm^−1^. Total 39623 reflections, 9703 unique (*R_int_* = 0.0728). Refinement of 7683 reflections (615 parameters) with *I* > 2σ(*I*) converged at final *R*_1_ = 0.0739 (*R*_1_ all data = 0.0962), *wR*_2_ = 0.2154 (*wR*2 all data = 0.2374), gof = 1.133. CCDC 2009747.

## 4. Conclusions

We have described the synthesis of compounds **8** and **9**, extending the series of known 4′-(4-*n*-alkyloxyphenyl)-3,2′:6′,3″-terpyridines. The compounds have been characterized by NMR, IR and absorption spectroscopies and mass spectrometry, and also by single-crystal X-ray crystallography. The structures of **6** and **7**, which possess shorter *n*-alkyloxy chains than **8** and **9**, have also been determined. At the molecular level, the structures of **6**, **7**, **8** and **9** are similar except for the conformation of the 3,2′:6′,3″-tpy which switches from *trans*,*trans* in **6** and **7** (conformation **I** in Scheme 1) to *cis*,*trans* in **8** and **9** (conformation **II**). In all the compounds, the *n*-alkyloxy chain is in an extended conformation. The solid-state structures of **6** and **7** consist of interwoven pairs of supramolecular 2D-sheets; each sheet features C–H...N hydrogen-bonded interactions and van der Waals interaction between pairs of *n*-hexyloxy (in **6**) or *n*-heptyloxy (in **7**) chains. The *n*-alkyloxy chains are threaded through cavities in an adjacent sheet to produce the woven-sheet motifs. On going from **6** and **7**, to **8** and **9**, the lengthening of the *n*-alkyloxy chains leads to significant changes in the packing interactions with a more dominant role for van der Waals interactions between adjacent *n*-alkyloxy chains and C–H_methylene_...π interactions. The structures of **8** and **9** comprise 2D-sheets supported by a combination of C–H...N and C–H...O weak hydrogen bonds, and van der Waals interactions between *n*-alkyloxy chains. The switch in 3,2′:6′,3″-tpy conformation is intimately associated with the network of C–H...N weak hydrogen bonds in the supramolecular assembly.

## Figures and Tables

**Table 1:** Conformations of previously reported free 3,2′:6′,3″-tpy ligand structures.

CSD Refcode	Crystallographically Independent 3,2′:6′,3″-tpy Units ^a^	Conformation(s)	Reference
TOQXUV	1	**I**	[20]
TOQYEG	1	**III**	[20]
BEPPUK	1	**I**	[21]
COJBEL, COJBEL01	4	**I ×** 2; **II ×** 2	[22]
FOBWOL	2	**I ×** 2	[23]
NEFVEC	1	**III**	[19]
SEPSAK	1	**I**	[24]

^a^ In COJBEL and COJBEL01, each molecule contains two 3,2′:6′,3″-tpy domains.

**Table 2:** Absorption maxima in the solution UV-VIS spectra of **6**, **8** and **9** (MeCN, 5 × 10^−5^ mol·dm^−3^ for **6** and **8**, 2 × 10^−5^ mol·dm^−3^ for **9**).

Compound	λ_max_/nm (ε/dm^3^·mol^−1^·cm^−1^)
**6**	228 (31,550), 270 (35,480)
**8**	227 (31,290), 271 (33,350)
**9**	227 (28,910), 273 (31,350)

**Table 3:** Selected structural parameters in compounds **6**–**9**; there are two crystallographic independent molecules of each compound.

Compound	Conformation ^a^	Angle between the Least Squares Planes of Pairs of Aromatic Rings/Deg		
		Pyridine outer/central	Pyridine outer/central	Pyridine central/phenylene
**6** molecule 1	**I**	13.0	14.3	19.0
**6** molecule 2	**I**	16.0	14.2	24.4
**7** molecule 1	**I**	18.2	9.0	15.5
**7** molecule 2	**I**	7.8	14.6	23.1
**8** molecule 1	**II**	16.8	5.7	20.6
**8** molecule 2	**II**	19.3	4.1	27.8
**9** molecule 1	**II**	5.9	7.2	20.2
**9** molecule 2	**II**	3.1	7.1	27.1

^a^ Conformations are defined in Scheme 1.

## Supplementary Materials

The following are available online. Figure S1. ESI mass spectrum of compound **8**. Figure S2. ESI mass spectrum of compound **9**. Figure S3. ^1^H NMR spectrum (500 MHz, CDCl_3_, 298 K) of compound **8**. Scale: δ/ppm. * = residual CHCl_3_. Figure S4. ^1^H NMR spectrum (500 MHz, CDCl_3_, 298 K) of compound **9**. Scale: δ/ppm. * = residual CHCl_3_. Figure S5. ^13^C{^1^H} NMR spectrum (126 MHz, CDCl_3_, 298 K) of compound **8**. Scale: δ/ppm. * = CDCl_3_. Figure S6. ^13^C{^1^H} NMR spectrum (126 MHz, CDCl_3_, 298 K) of compound **8**. Scale: δ/ppm. * = CDCl_3_. Figure S7. HMQC spectrum of compound 8 (^1^H 500 MHz, ^13^C 126 MHz, CDCl_3_, 298 K). Scale: δ/ ppm. * = residual CHCl_3_ or CDCl_3_. Figure S8. HMQC spectrum of compound **9** (^1^H 500 MHz, ^13^C 126 MHz, CDCl_3_, 298 K). Scale: δ/ppm. * = residual CHCl_3_ or CDCl_3_. Figure S9. HMBC spectrum of compound **9** (^1^H 500 MHz, ^13^C 126 MHz, CDCl_3_, 298 K). Scale: δ/ppm. * = residual CHCl_3_ or CDCl_3_. Figure S10. HMBC spectrum of compound **9** (^1^H 500 MHz, ^13^C 126 MHz, CDCl_3_, 298 K). Scale: δ/ppm. * = residual CHCl_3_ or CDCl_3_. Figure S11. Solid-state FT-IR spectrum of compound 8. Figure S12. Solid-state FT-IR spectrum of compound **9**. Figure S13. Head-to-tail (centrosymmetric) packing of 3,2’:6’,3"-tpy units in **6**. Figure S14. Part of two layers in the crystal lattice of 8 in which C–Hmethylene... *π* interactions contribite to the packing. Figure S15. Space-filling representation of the packing diagram shown in the manuscript in Figure 8. Figure S16. Space-filling representation of the packing diagram shown in the manuscript in Figure 10. Figures S1–S12: Mass spectra, NMR spectra and IR spectra of compounds **8** and **9**; Figures S13–S16: Additional packing diagrams.
