# Supplementary material for: Switching the Conformation of 3,2′:6′,3″-tpy Domains in 4′-(4-n-Alkyloxyphenyl)-3,2′:6′,3″-Terpyridines
Source: Molecules. 2020 Jul 10;25(14):3162. doi: 10.3390/molecules25143162 (PMC7397000; doi:10.3390/molecules25143162)

checkCIF/PLATON report

Structure factors have been supplied for datablock(s) rod020_300k

THIS REPORT IS FOR GUIDANCE ONLY. IF USED AS PART OF A REVIEW PROCEDURE FOR PUBLICATION, IT SHOULD NOT REPLACE THE EXPERTISE OF AN EXPERIENCED CRYSTALLOGRAPHIC REFEREE.

No syntax errors found. CIF dictionary Interpreting this report

Datablock: rod020_300k

```
Bond precision:  C-C = 0.0023 A          Wavelength=1.34143

Cell:            a=18.8773(3)     b=11.3154(2)       c=21.6453(3)
                 alpha=90         beta=105.985(1)    gamma=90
Temperature:     300 K
```

	Calculated	Reported
Volume	4444.75(12)	4444.75(12)
Space group	P 21/n	P 1 21/n 1
Hall group	-P 2yn	-P 2yn
Moiety formula	C27 H27 N3 O	C27 H27 N3 O
Sum formula	C27 H27 N3 O	C27 H27 N3 O
Mr	409.52	409.51
Dx,g cm-3	1.224	1.224
Z	8	8
Mu (mm-1)	0.375	0.378
F000	1744.0	1744.0
F000’	1747.53	
h,k,lmax	23,14,27	23,14,27
Nref	9149	8962
Tmin,Tmax	0.896,0.934	0.290,0.992
Tmin’	0.893	

```
Correction method= # Reported T Limits: Tmin=0.290 Tmax=0.992
AbsCorr = MULTI-SCAN

Data completeness= 0.980          Theta(max)= 57.132

R(reflections)= 0.0537( 7622)     wR2(reflections)= 0.1702( 8962)

S = 1.055                  Npar= 562
```

The following ALERTS were generated. Each ALERT has the format
test-name_ALERT_alert-type_alert-level.
Click on the hyperlinks for more details of the test.

Alert level C

```
PLAT241_ALERT_2_C High   'MainMol' Ueq as Compared to Neighbors of        C46 Check
PLAT911_ALERT_3_C Missing FCF Refl Between Thmin & STh/L=    0.600         16 Report
PLAT918_ALERT_3_C Reflection(s) with I(obs) much Smaller I(calc) .          4 Check
PLAT939_ALERT_3_C Large Value of Not (SHELXL) Weight Optimized S .      10.90 Check
```

Alert level G

```
ABSMU01_ALERT_1_G  Calculation of _exptl_absorpt_correction_mu
                not performed for this radiation type.
PLAT910_ALERT_3_G Missing # of FCF Reflection(s) Below Theta(Min).          1 Note
PLAT912_ALERT_4_G Missing # of FCF Reflections Above STh/L=  0.600        171 Note
PLAT978_ALERT_2_G Number C-C Bonds with Positive Residual Density.          6 Info
PLAT984_ALERT_1_G The  C-f'=    0.0148 Deviates from the B&C-Value     0.0137 Check
PLAT984_ALERT_1_G The  N-f'=    0.0253 Deviates from the B&C-Value     0.0241 Check
PLAT984_ALERT_1_G The  O-f'=    0.0412 Deviates from the B&C-Value     0.0389 Check
```

---

0 **ALERT level A** = Most likely a serious problem - resolve or explain
0 **ALERT level B** = A potentially serious problem, consider carefully
4 **ALERT level C** = Check. Ensure it is not caused by an omission or oversight
7 **ALERT level G** = General information/check it is not something unexpected

4 ALERT type 1 CIF construction/syntax error, inconsistent or missing data
2 ALERT type 2 Indicator that the structure model may be wrong or deficient
4 ALERT type 3 Indicator that the structure quality may be low
1 ALERT type 4 Improvement, methodology, query or suggestion
0 ALERT type 5 Informative message, check

It is advisable to attempt to resolve as many as possible of the alerts in all categories. Often the minor alerts point to easily fixed oversights, errors and omissions in your CIF or refinement strategy, so attention to these fine details can be worthwhile. In order to resolve some of the more serious problems it may be necessary to carry out additional measurements or structure refinements. However, the purpose of your study may justify the reported deviations and the more serious of these should normally be commented upon in the discussion or experimental section of a paper or in the "special_details" fields of the CIF. checkCIF was carefully designed to identify outliers and unusual parameters, but every test has its limitations and alerts that are not important in a particular case may appear. Conversely, the absence of alerts does not guarantee there are no aspects of the results needing attention. It is up to the individual to critically assess their own results and, if necessary, seek expert advice.

Publication of your CIF in IUCr journals

A basic structural check has been run on your CIF. These basic checks will be run on all CIFs submitted for publication in IUCr journals (*Acta Crystallographica*, *Journal of Applied Crystallography*, *Journal of Synchrotron Radiation*); however, if you intend to submit to *Acta Crystallographica Section C* or *E* or *IUCrData*, you should make sure that full publication checks are run on the final version of your CIF prior to submission.

Publication of your CIF in other journals

Please refer to the *Notes for Authors* of the relevant journal for any special instructions relating to CIF submission.

PLATON version of 03/05/2019; check.def file version of 29/04/2019

Datablock rod020_300k - ellipsoid plot

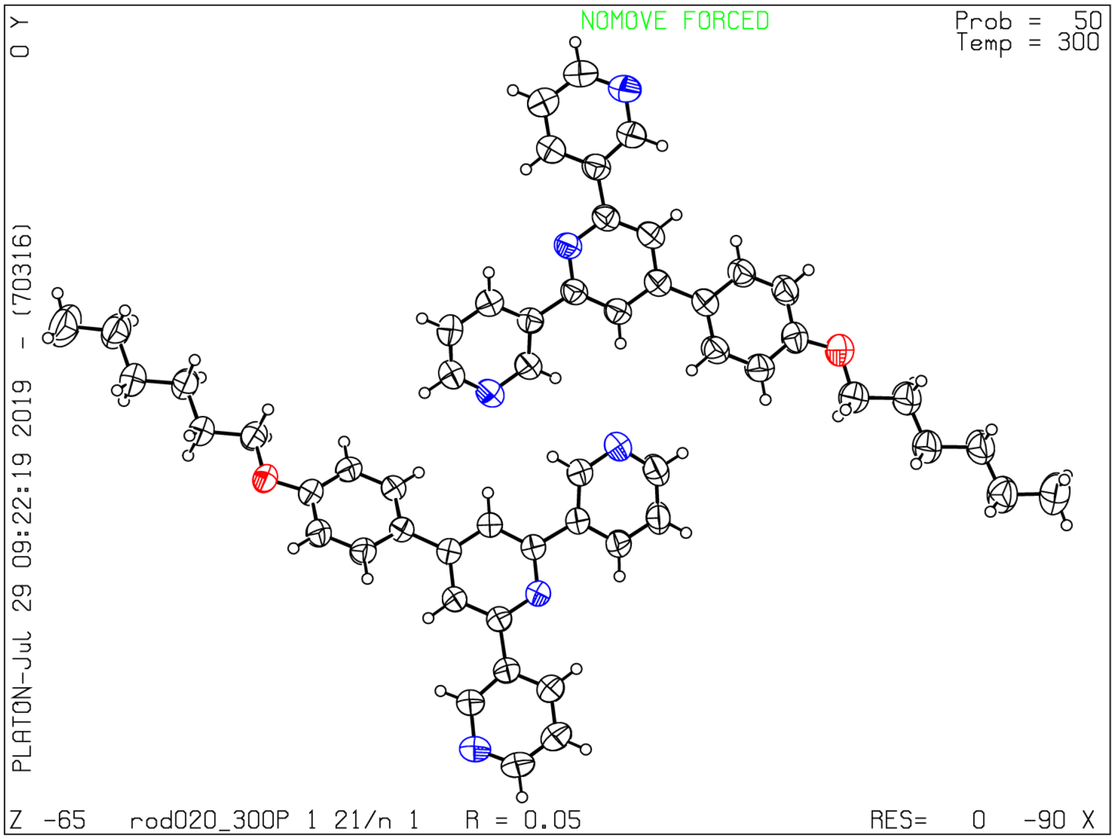

Supplement: Supplementary file 1 [file molecules-25-03162-s001.zip › molecules-855758-supplementary-proof - ORIGINAL/ROD020_checkcif.pdf]
