# Supplementary material for: Switching the Conformation of 3,2′:6′,3″-tpy Domains in 4′-(4-n-Alkyloxyphenyl)-3,2′:6′,3″-Terpyridines
Source: Molecules. 2020 Jul 10;25(14):3162. doi: 10.3390/molecules25143162 (PMC7397000; doi:10.3390/molecules25143162)

checkCIF/PLATON report

Structure factors have been supplied for datablock(s) rod028_130k

THIS REPORT IS FOR GUIDANCE ONLY. IF USED AS PART OF A REVIEW PROCEDURE FOR PUBLICATION, IT SHOULD NOT REPLACE THE EXPERTISE OF AN EXPERIENCED CRYSTALLOGRAPHIC REFEREE.

No syntax errors found.   CIF dictionary   Interpreting this report

Datablock: rod028_130k

```
Bond precision:  C-C = 0.0021 A          Wavelength=1.54178

Cell:           a=23.1434(10)    b=10.8165(5)       c=18.7860(9)
                alpha=90         beta=108.115(3)    gamma=90
Temperature:    130 K

                Calculated                Reported
Volume          4469.6(4)                 4469.6(4)
Space group     P 21/c                    P 1 21/c 1
Hall group      -P 2ybc                   -P 2ybc
Moiety formula  C28 H29 N3 O              C28 H29 N3 O
Sum formula     C28 H29 N3 O              C28 H29 N3 O
Mr              423.54                    423.54
Dx,g cm-3       1.259                     1.259
Z               8                         8
Mu (mm-1)       0.601                     0.601
F000            1808.0                    1808.0
F000'           1812.87
h,k,lmax        28,13,22                  27,13,22
Nref            8525                      8165
Tmin,Tmax       0.897,0.942               0.647,0.753
Tmin'           0.887

Correction method= # Reported T Limits: Tmin=0.647 Tmax=0.753
AbsCorr = MULTI-SCAN

Data completeness= 0.958          Theta(max)= 70.289

R(reflections)= 0.0429( 6195)     wR2(reflections)= 0.1205( 8165)

S = 1.025                  Npar= 579
```

The following ALERTS were generated. Each ALERT has the format
test-name_ALERT_alert-type_alert-level.
Click on the hyperlinks for more details of the test.

Alert level C

```
PLAT911_ALERT_3_C Missing FCF Refl Between Thmin & STh/L=    0.600         61 Report
```

Alert level G

```
PLAT912_ALERT_4_G Missing # of FCF Reflections Above STh/L=  0.600        290 Note
PLAT941_ALERT_3_G Average HKL Measurement Multiplicity ...........        3.7 Low
PLAT978_ALERT_2_G Number C-C Bonds with Positive Residual Density.         10 Info
PLAT992_ALERT_5_G Repd & Actual _reflns_number_gt Values Differ by          5 Check
```

```
   0 ALERT level A = Most likely a serious problem - resolve or explain
   0 ALERT level B = A potentially serious problem, consider carefully
   1 ALERT level C = Check. Ensure it is not caused by an omission or oversight
   4 ALERT level G = General information/check it is not something unexpected

   0 ALERT type 1 CIF construction/syntax error, inconsistent or missing data
   1 ALERT type 2 Indicator that the structure model may be wrong or deficient
   2 ALERT type 3 Indicator that the structure quality may be low
   1 ALERT type 4 Improvement, methodology, query or suggestion
   1 ALERT type 5 Informative message, check
```

It is advisable to attempt to resolve as many as possible of the alerts in all categories. Often the minor alerts point to easily fixed oversights, errors and omissions in your CIF or refinement strategy, so attention to these fine details can be worthwhile. In order to resolve some of the more serious problems it may be necessary to carry out additional measurements or structure refinements. However, the purpose of your study may justify the reported deviations and the more serious of these should normally be commented upon in the discussion or experimental section of a paper or in the "special_details" fields of the CIF. checkCIF was carefully designed to identify outliers and unusual parameters, but every test has its limitations and alerts that are not important in a particular case may appear. Conversely, the absence of alerts does not guarantee there are no aspects of the results needing attention. It is up to the individual to critically assess their own results and, if necessary, seek expert advice.

Publication of your CIF in IUCr journals

A basic structural check has been run on your CIF. These basic checks will be run on all CIFs submitted for publication in IUCr journals (*Acta Crystallographica*, *Journal of Applied Crystallography*, *Journal of Synchrotron Radiation*); however, if you intend to submit to *Acta Crystallographica Section C* or *E* or *IUCrData*, you should make sure that full publication checks are run on the final version of your CIF prior to submission.

Publication of your CIF in other journals

Please refer to the *Notes for Authors* of the relevant journal for any special instructions relating to CIF submission.

PLATON version of 04/06/2020; check.def file version of 02/06/2020

Datablock rod028_130k - ellipsoid plot

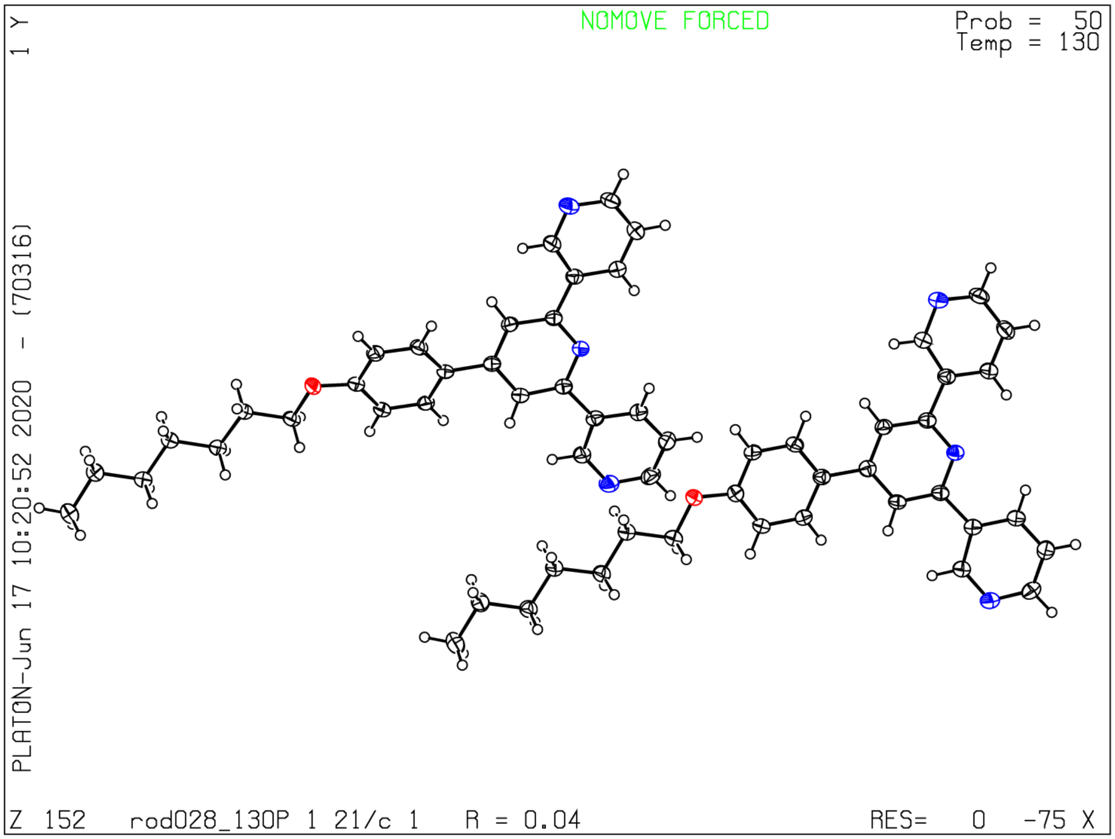

Supplement: Supplementary file 1 [file molecules-25-03162-s001.zip › molecules-855758-supplementary-proof - ORIGINAL/ROD028_130K_checkcif.pdf]
