# Supplementary material for: Switching the Conformation of 3,2′:6′,3″-tpy Domains in 4′-(4-n-Alkyloxyphenyl)-3,2′:6′,3″-Terpyridines
Source: Molecules. 2020 Jul 10;25(14):3162. doi: 10.3390/molecules25143162 (PMC7397000; doi:10.3390/molecules25143162)

checkCIF/PLATON report

Structure factors have been supplied for datablock(s) rod066_130k

THIS REPORT IS FOR GUIDANCE ONLY. IF USED AS PART OF A REVIEW PROCEDURE FOR PUBLICATION, IT SHOULD NOT REPLACE THE EXPERTISE OF AN EXPERIENCED CRYSTALLOGRAPHIC REFEREE.

No syntax errors found.   CIF dictionary   Interpreting this report

Datablock: rod066_130k

```
Bond precision:  C-C = 0.0041 A          Wavelength=1.34143

Cell:           a=11.6102(4)      b=13.9680(5)     c=16.4766(6)
                alpha=95.198(3)   beta=99.538(3)   gamma=108.195(3)
Temperature:    130 K

                Calculated                  Reported
Volume          2474.28(16)                 2474.27(16)
Space group     P -1                        P -1
Hall group      -P 1                        -P 1
Moiety formula  C30 H34 N3 O                C30 H34 N3 O
Sum formula     C30 H34 N3 O                C30 H34 N3 O
Mr              452.60                      452.60
Dx,g cm-3       1.215                       1.215
Z               4                           4
Mu (mm-1)       0.365                       0.369
F000            972.0                       972.0
F000'           973.92
h,k,lmax        14,17,20                    14,17,20
Nref            10041                       9703
Tmin,Tmax       0.932,0.960                 0.990,0.995
Tmin'           0.925

Correction method= # Reported T Limits: Tmin=0.990 Tmax=0.995
AbsCorr = MULTI-SCAN

Data completeness= 0.966          Theta(max)= 56.713

R(reflections)= 0.0766( 7683)     wR2(reflections)= 0.2458( 9703)

S = 1.115                  Npar= 615
```

The following ALERTS were generated. Each ALERT has the format
test-name_ALERT_alert-type_alert-level.
Click on the hyperlinks for more details of the test.

Alert level B

```
PLAT414_ALERT_2_B Short Intra D-H..H-X       H2        ..H18             1.87 Ang.
                                                       x,y,z  =      1_555 Check
PLAT414_ALERT_2_B Short Intra D-H..H-X       H2        ..H21             1.83 Ang.
                                                       x,y,z  =      1_555 Check
PLAT414_ALERT_2_B Short Intra D-H..H-X       H5        ..H39             1.87 Ang.
                                                       x,y,z  =      1_555 Check
PLAT414_ALERT_2_B Short Intra D-H..H-X       H5        ..H42             1.83 Ang.
                                                       x,y,z  =      1_555 Check
PLAT934_ALERT_3_B Number of (Iobs-Icalc)/Sigma(W) > 10 Outliers ..          7 Check
```

Alert level C

```
DIFMN02_ALERT_2_C  The minimum difference density is < -0.1*ZMAX*0.75
            _refine_diff_density_min given =     -0.606
            Test value =     -0.600
DIFMN03_ALERT_1_C  The minimum difference density is < -0.1*ZMAX*0.75
            The relevant atom site should be identified.
PLAT098_ALERT_2_C Large Reported Min.  (Negative) Residual Density      -0.61 eA-3
PLAT112_ALERT_2_C ADDSYM Detects New (Pseudo) Symm. Elem       c/2         88 %Fit
PLAT340_ALERT_3_C Low Bond Precision on  C-C Bonds ...............     0.0041 Ang.
PLAT420_ALERT_2_C D-H Without Acceptor       N2        --H2       .     Please Check
PLAT420_ALERT_2_C D-H Without Acceptor       N5        --H5       .     Please Check
PLAT906_ALERT_3_C Large K Value in the Analysis of Variance ......     15.401 Check
PLAT906_ALERT_3_C Large K Value in the Analysis of Variance ......      2.858 Check
PLAT911_ALERT_3_C Missing FCF Refl Between Thmin & STh/L=    0.600         53 Report
PLAT918_ALERT_3_C Reflection(s) with I(obs) much Smaller I(calc) .          3 Check
PLAT939_ALERT_3_C Large Value of Not (SHELXL) Weight Optimized S .      10.60 Check
PLAT976_ALERT_2_C Check Calcd Resid. Dens.  0.91A   From N5             -0.60 eA-3
PLAT976_ALERT_2_C Check Calcd Resid. Dens.  0.86A   From N2             -0.56 eA-3
PLAT977_ALERT_2_C Check Negative Difference Density on H2               -0.56 eA-3
PLAT977_ALERT_2_C Check Negative Difference Density on H5               -0.52 eA-3
```

Alert level G

```
ABSMU01_ALERT_1_G  Calculation of _exptl_absorpt_correction_mu
                not performed for this radiation type.
PLAT007_ALERT_5_G Number of Unrefined Donor-H Atoms ..............          2 Report
PLAT072_ALERT_2_G SHELXL First  Parameter in WGHT  Unusually Large       0.10 Report
PLAT154_ALERT_1_G The s.u.'s on the Cell Angles are Equal ..(Note)      0.003 Degree
PLAT912_ALERT_4_G Missing # of FCF Reflections Above STh/L=  0.600        285 Note
PLAT933_ALERT_2_G Number of OMIT Records in Embedded .res File ...          3 Note
PLAT978_ALERT_2_G Number C-C Bonds with Positive Residual Density.          5 Info
PLAT984_ALERT_1_G The  C-f'=    0.0148 Deviates from the B&C-Value     0.0137 Check
PLAT984_ALERT_1_G The  N-f'=    0.0253 Deviates from the B&C-Value     0.0241 Check
PLAT984_ALERT_1_G The  O-f'=    0.0412 Deviates from the B&C-Value     0.0389 Check
PLAT992_ALERT_5_G Repd & Actual _reflns_number_gt Values Differ by          1 Check
```

```
   0 ALERT level A = Most likely a serious problem - resolve or explain
   5 ALERT level B = A potentially serious problem, consider carefully
  16 ALERT level C = Check. Ensure it is not caused by an omission or oversight
  11 ALERT level G = General information/check it is not something unexpected

   6 ALERT type 1 CIF construction/syntax error, inconsistent or missing data
  16 ALERT type 2 Indicator that the structure model may be wrong or deficient
   7 ALERT type 3 Indicator that the structure quality may be low
   1 ALERT type 4 Improvement, methodology, query or suggestion
   2 ALERT type 5 Informative message, check
```

Validation response form

Please find below a validation response form (VRF) that can be filled in and pasted into your CIF.

```
# start Validation Reply Form
_vrf_DIFMN02_rod066_130k
;
PROBLEM: The minimum difference density is < -0.1*ZMAX*0.75
RESPONSE: ...
;
_vrf_DIFMN03_rod066_130k
;
PROBLEM: The minimum difference density is < -0.1*ZMAX*0.75
RESPONSE: ...
;
_vrf_PLAT098_rod066_130k
;
PROBLEM: Large Reported Min.  (Negative) Residual Density      -0.61 eA-3
RESPONSE: ...
;
_vrf_PLAT112_rod066_130k
;
PROBLEM: ADDSYM Detects New (Pseudo) Symm. Elem       c/2         88 %Fit
RESPONSE: ...
;
_vrf_PLAT340_rod066_130k
;
PROBLEM: Low Bond Precision on  C-C Bonds ...............     0.0041 Ang.
RESPONSE: ...
;
_vrf_PLAT420_rod066_130k
;
PROBLEM: D-H Without Acceptor       N2       --H2       .     Please Check
RESPONSE: ...
;
_vrf_PLAT906_rod066_130k
;
PROBLEM: Large K Value in the Analysis of Variance ......     15.401 Check
RESPONSE: ...
;
_vrf_PLAT911_rod066_130k
;
PROBLEM: Missing FCF Refl Between Thmin & STh/L=    0.600         53 Report
RESPONSE: ...
;
_vrf_PLAT918_rod066_130k
;
PROBLEM: Reflection(s) with I(obs) much Smaller I(calc) .          3 Check
RESPONSE: ...
;
_vrf_PLAT939_rod066_130k
;
PROBLEM: Large Value of Not (SHELXL) Weight Optimized S .      10.60 Check
RESPONSE: ...
;
_vrf_PLAT976_rod066_130k
;
PROBLEM: Check Calcd Resid. Dens.  0.91A   From N5             -0.60 eA-3
RESPONSE: ...
;
_vrf_PLAT977_rod066_130k
```

```
;
PROBLEM: Check Negative Difference Density on H2               -0.56 eA-3
RESPONSE: ...
;
# end Validation Reply Form
```

It is advisable to attempt to resolve as many as possible of the alerts in all categories. Often the minor alerts point to easily fixed oversights, errors and omissions in your CIF or refinement strategy, so attention to these fine details can be worthwhile. In order to resolve some of the more serious problems it may be necessary to carry out additional measurements or structure refinements. However, the purpose of your study may justify the reported deviations and the more serious of these should normally be commented upon in the discussion or experimental section of a paper or in the "special_details" fields of the CIF. checkCIF was carefully designed to identify outliers and unusual parameters, but every test has its limitations and alerts that are not important in a particular case may appear. Conversely, the absence of alerts does not guarantee there are no aspects of the results needing attention. It is up to the individual to critically assess their own results and, if necessary, seek expert advice.

Publication of your CIF in IUCr journals

A basic structural check has been run on your CIF. These basic checks will be run on all CIFs submitted for publication in IUCr journals (*Acta Crystallographica*, *Journal of Applied Crystallography*, *Journal of Synchrotron Radiation*); however, if you intend to submit to *Acta Crystallographica Section C* or *E* or *IUCrData*, you should make sure that full publication checks are run on the final version of your CIF prior to submission.

Publication of your CIF in other journals

Please refer to the *Notes for Authors* of the relevant journal for any special instructions relating to CIF submission.

PLATON version of 07/08/2019; check.def file version of 30/07/2019

Datablock rod066_130k - ellipsoid plot

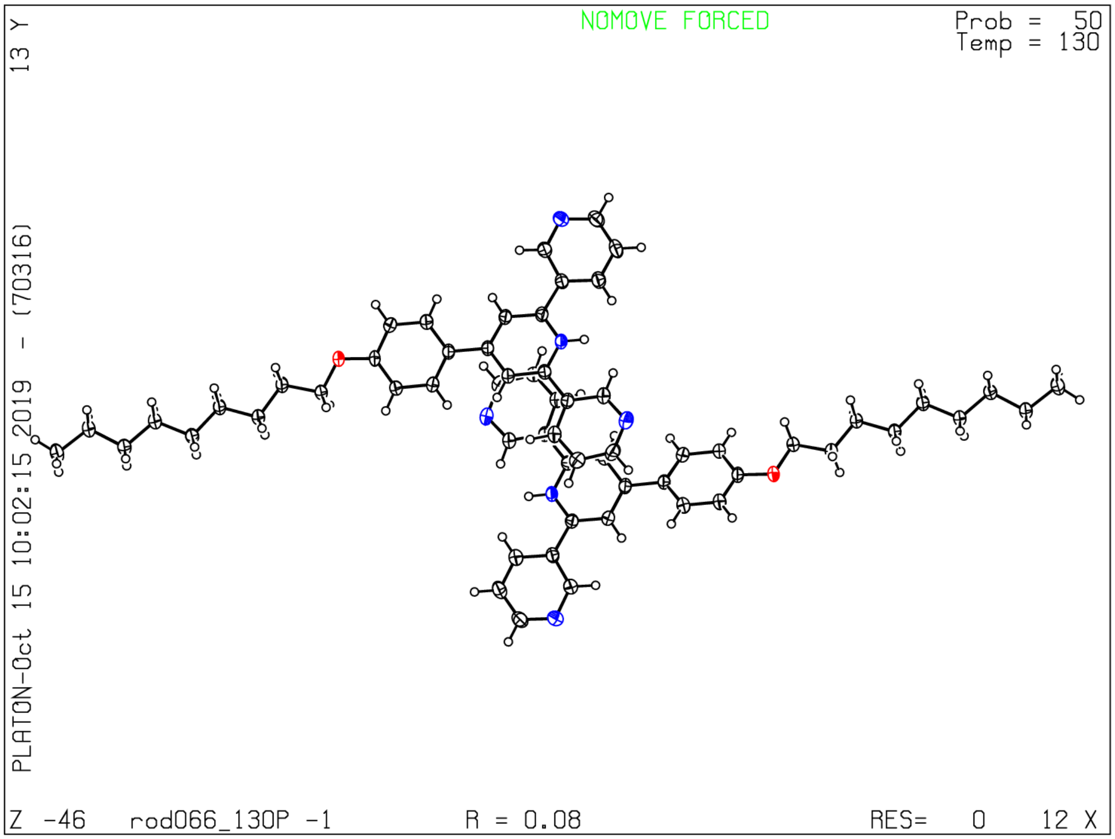

Supplement: Supplementary file 1 [file molecules-25-03162-s001.zip › molecules-855758-supplementary-proof - ORIGINAL/ROD066checkcif.pdf]
