# Supplementary material for: Switching the Conformation of 3,2′:6′,3″-tpy Domains in 4′-(4-n-Alkyloxyphenyl)-3,2′:6′,3″-Terpyridines
Source: Molecules. 2020 Jul 10;25(14):3162. doi: 10.3390/molecules25143162 (PMC7397000; doi:10.3390/molecules25143162)
Supplement: Supplementary file 1 [file molecules-25-03162-s001.zip › molecules-855758-supplementary-proof - ORIGINAL/Supporting Materials for.pdf]

Supporting Material for

Switching the conformation of 3,2':6',3''-tpy domains in 4'-(4-*n*-alkyloxyphenyl)-3,2':6',3''-terpyridine

Dalila Rocco [1], Alessandro Prescimone [1], Edwin C. Constable [1] and Catherine E. Housecroft [1,*]

[1] Department of Chemistry, University of Basel, BPR 1096, Mattenstrasse 24a, CH-4058 Basel, Switzerland; dalila.rocco@unibas.ch (D.R.); alessandro.prescimone@unibas.ch (A.P.); edwin.constable@unibas.ch (E.C.C.)

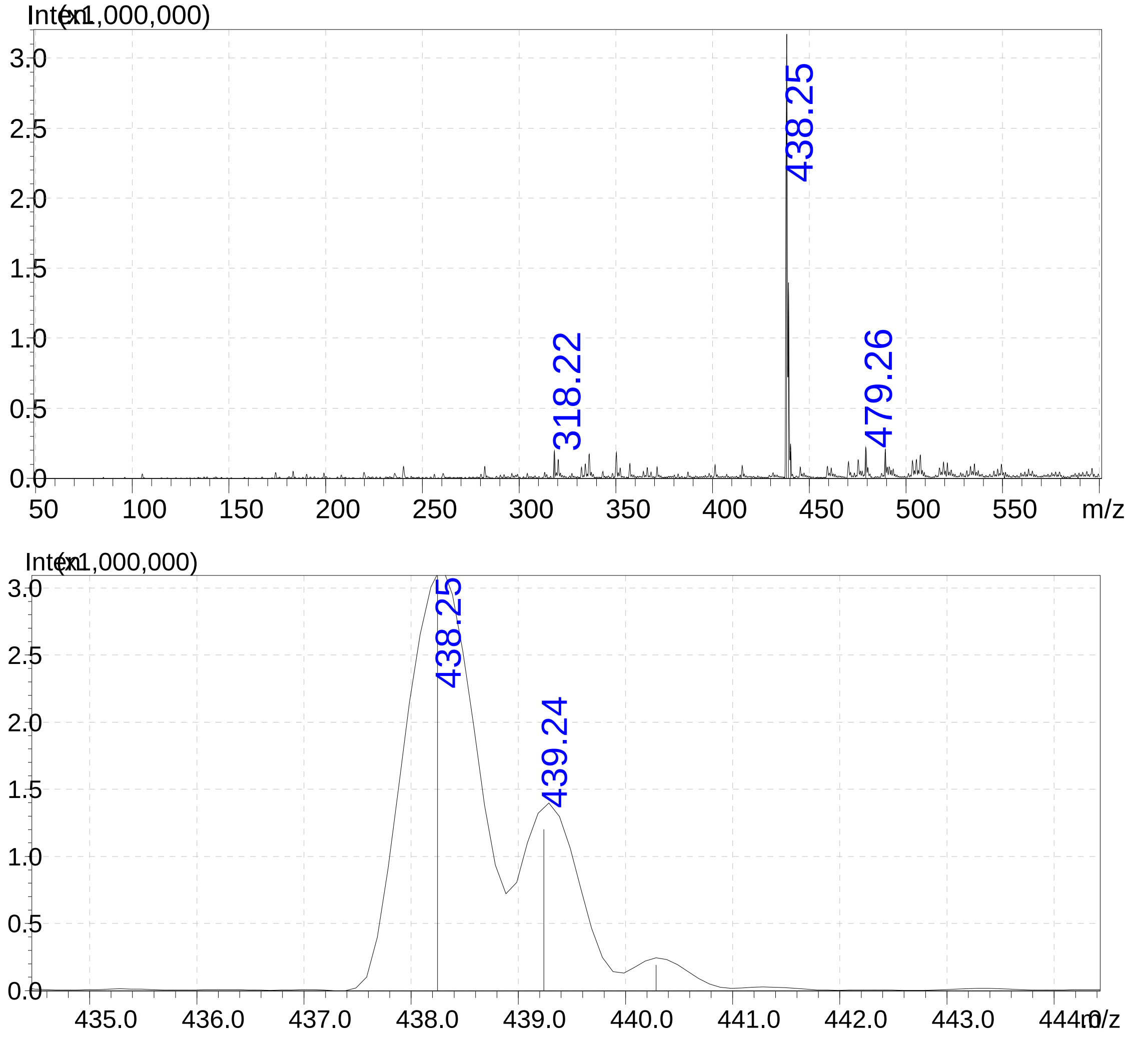

Figure S1. ESI mass spectrum of compound **8**.

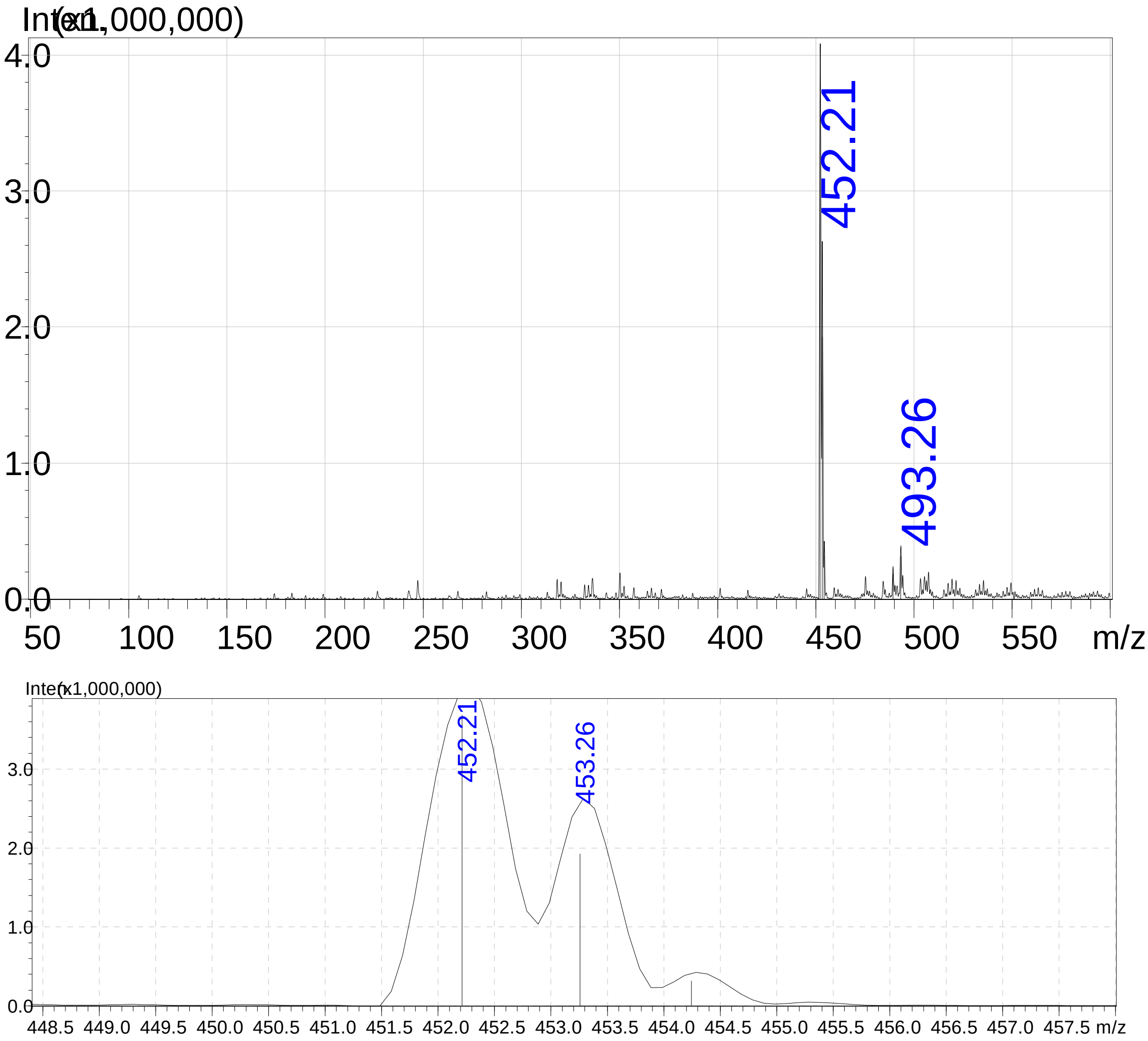

Figure S2. ESI mass spectrum of compound **9**.

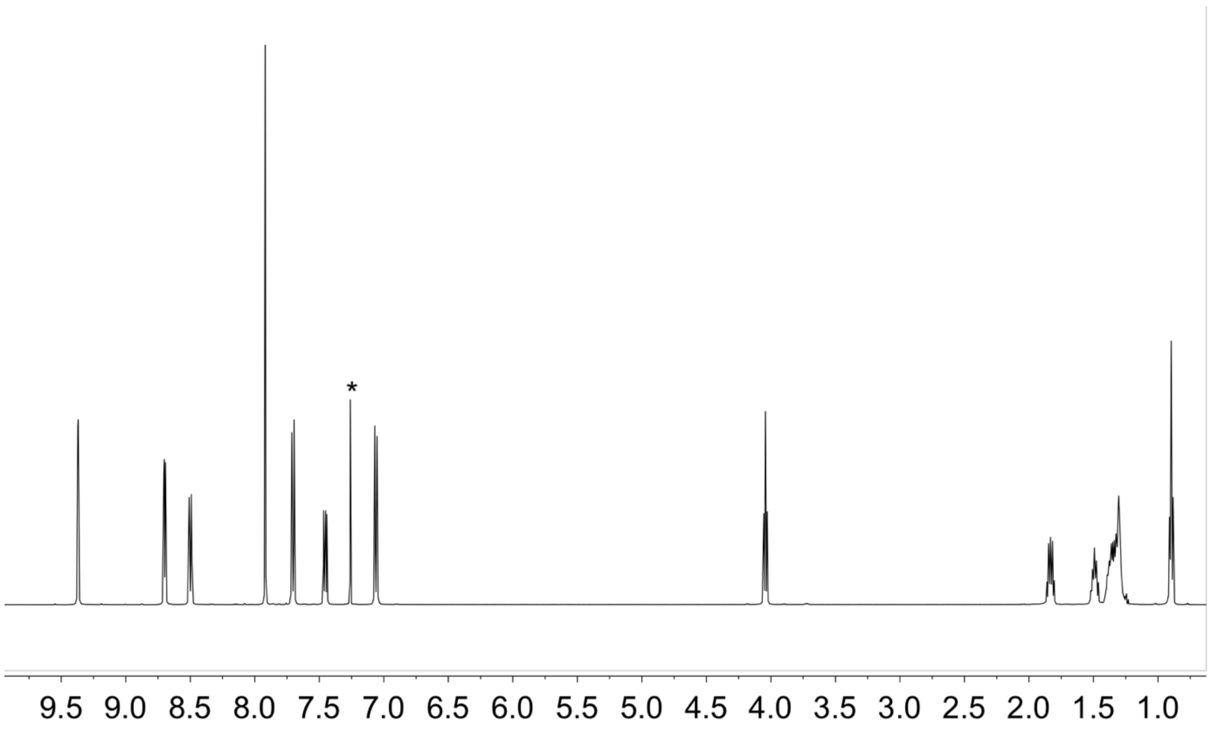

Figure S3. ^{1}H NMR spectrum (500 MHz, $CDCl_3$, 298 K) of compound **8**. Scale: δ / ppm. * = residual $CHCl_3$.

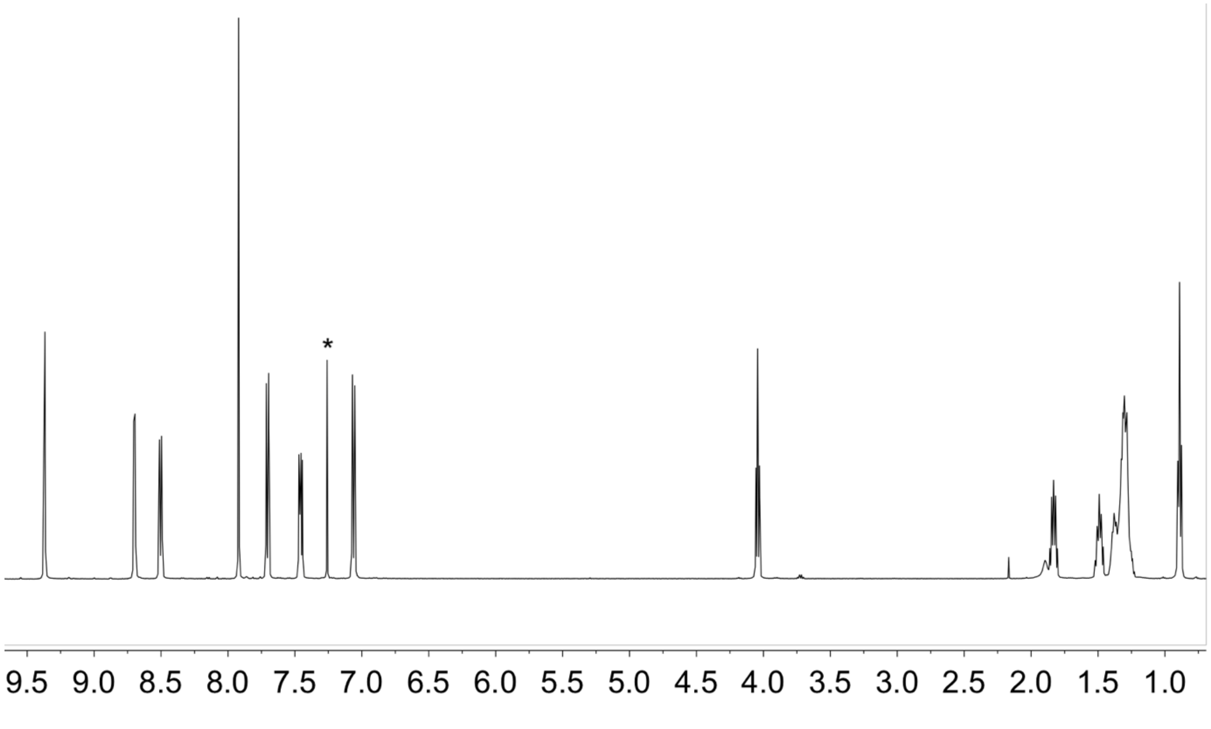

Figure S4. ^{1}H NMR spectrum (500 MHz, $CDCl_3$, 298 K) of compound **9**. Scale: δ / ppm. * = residual $CHCl_3$.

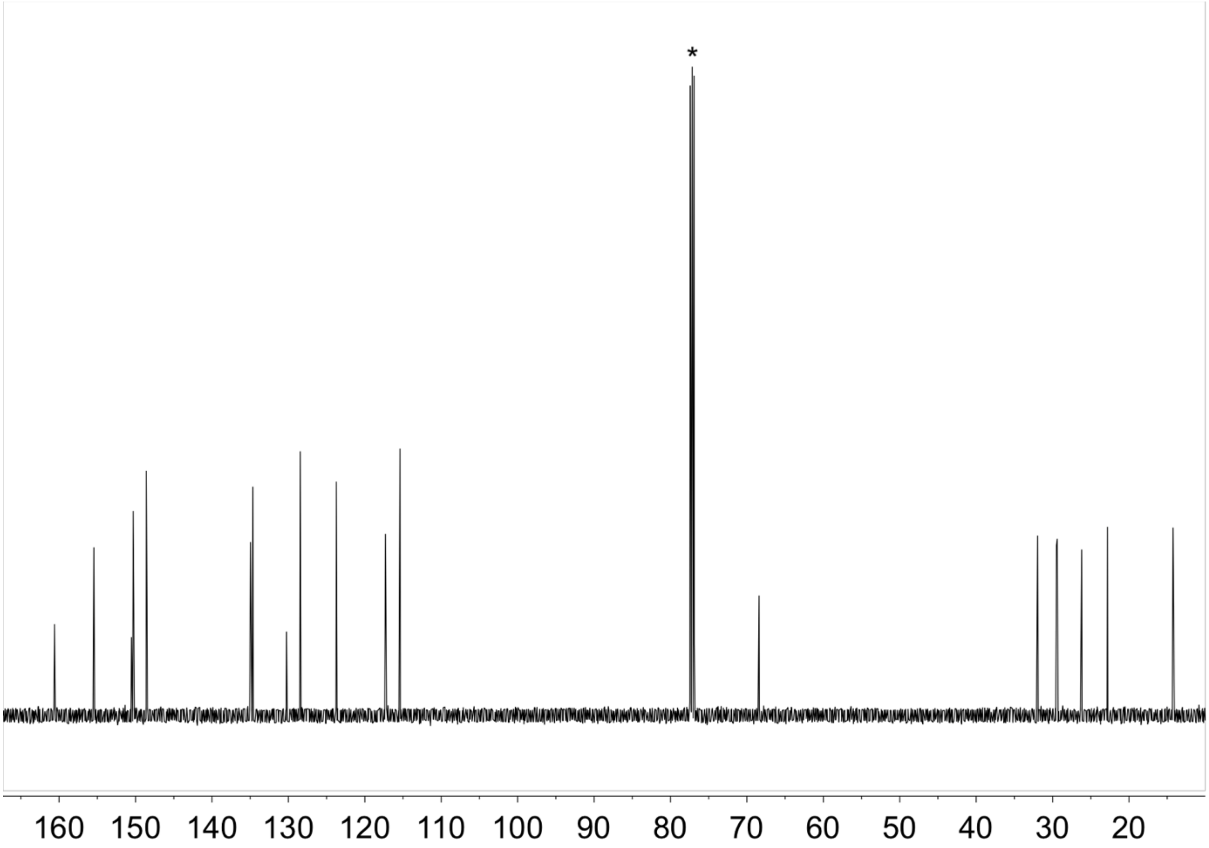

Figure S5. $^{13}C\{^{1}H\}$ NMR spectrum (126 MHz, $CDCl_3$, 298 K) of compound **8**. Scale: δ / ppm. * = $CDCl_3$.

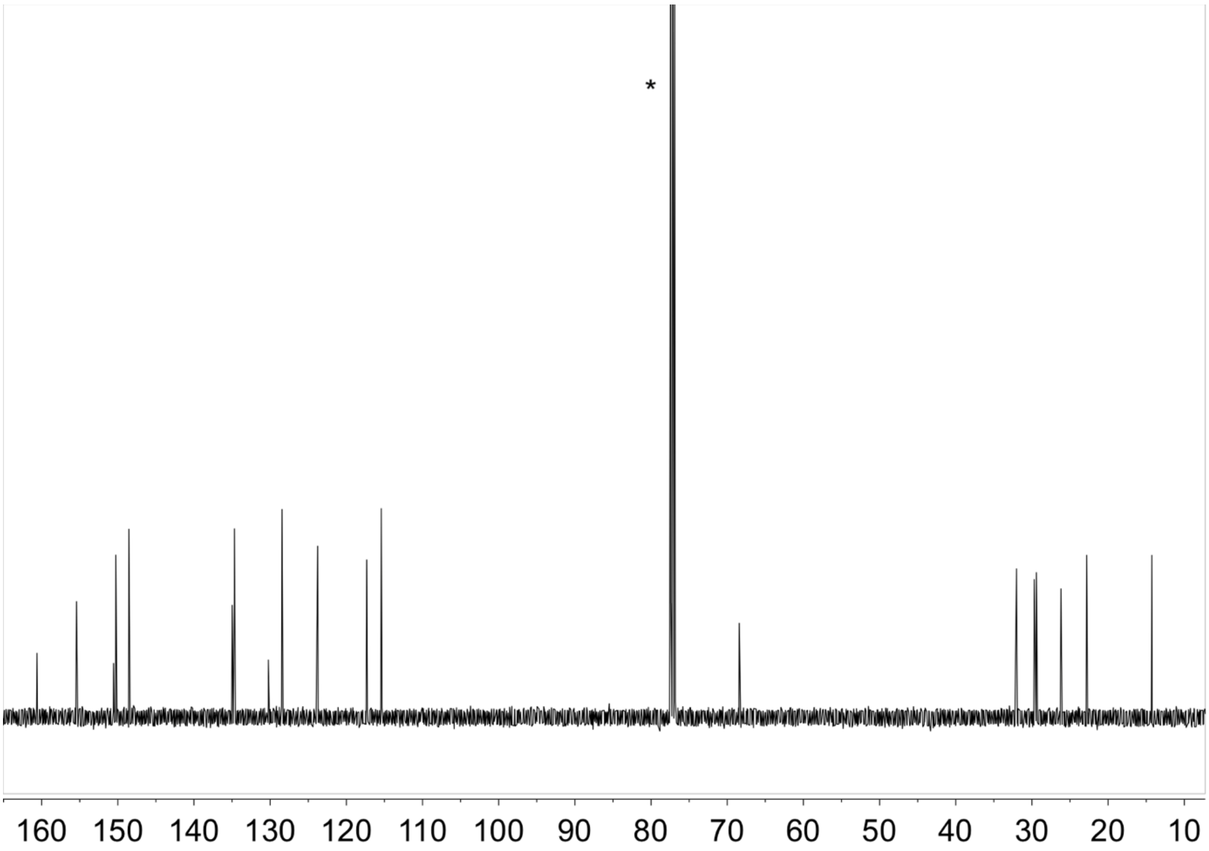

Figure S6. $^{13}C\{^{1}H\}$ NMR spectrum (126 MHz, $CDCl_3$, 298 K) of compound **8**. Scale: δ / ppm. * = $CDCl_3$.

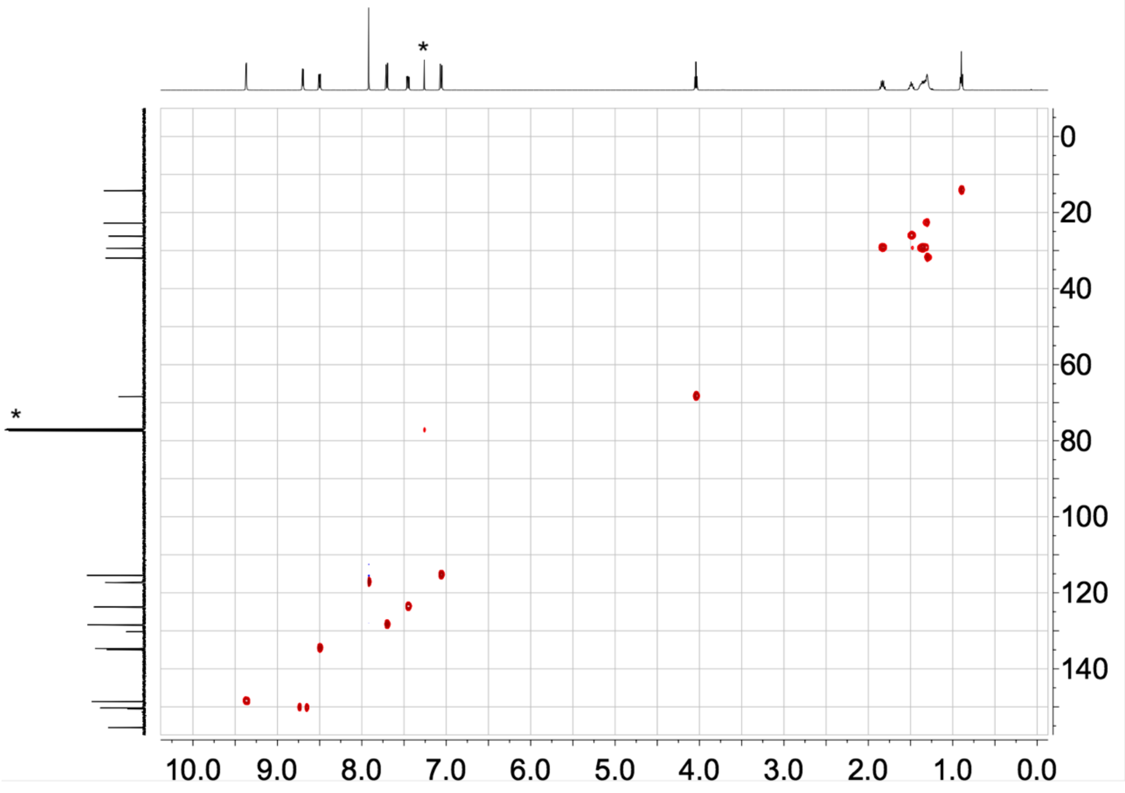

Figure S7. HMQC spectrum of compound **8** (^{1}H 500 MHz, ^{13}C 126 MHz, $CDCl_3$, 298 K). Scale: δ / ppm. * = residual $CHCl_3$ or $CDCl_3$.

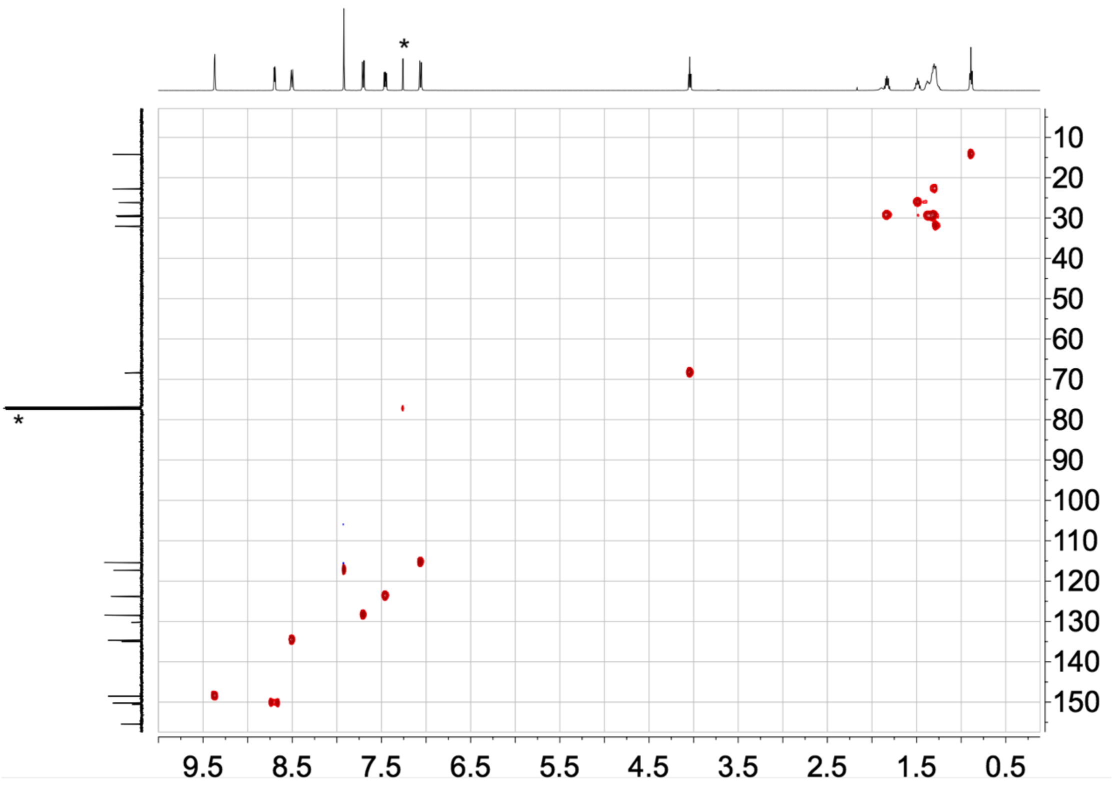

Figure S8. HMQC spectrum of compound **9** (^{1}H 500 MHz, ^{13}C 126 MHz, $CDCl_3$, 298 K). Scale: δ / ppm. * = residual $CHCl_3$ or $CDCl_3$.

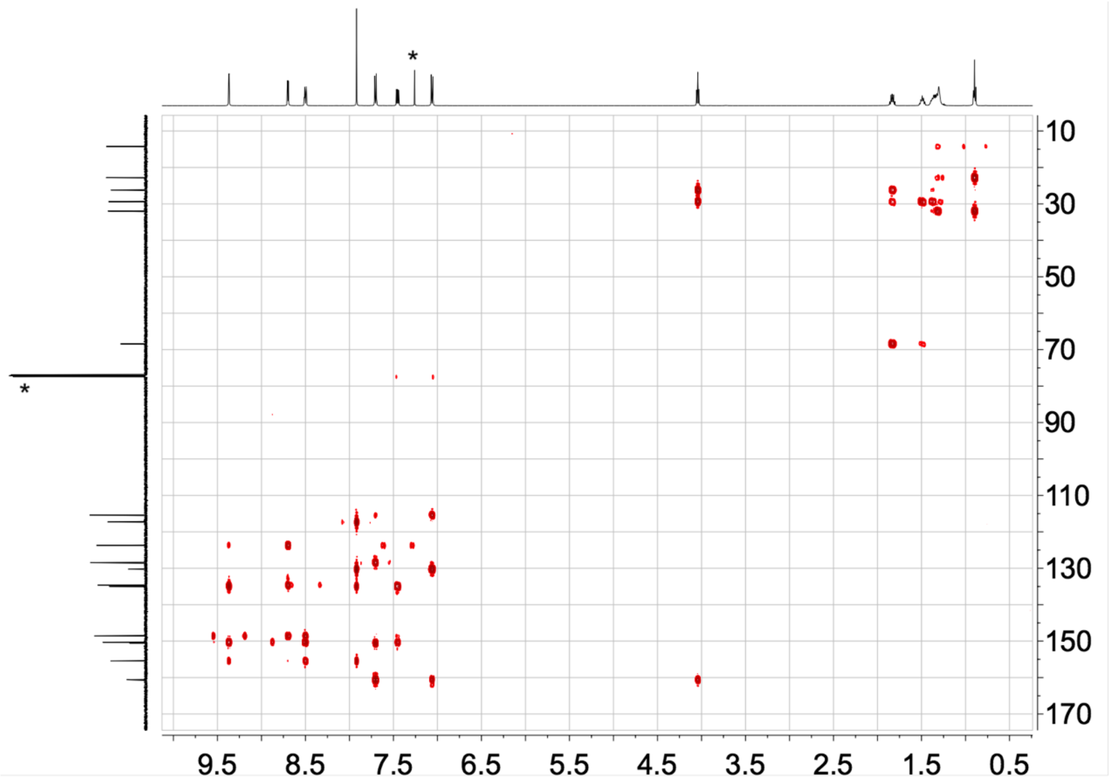

Figure S9. HMBC spectrum of compound **9** (^{1}H 500 MHz, ^{13}C 126 MHz, $CDCl_3$, 298 K). Scale: δ / ppm. * = residual $CHCl_3$ or $CDCl_3$.

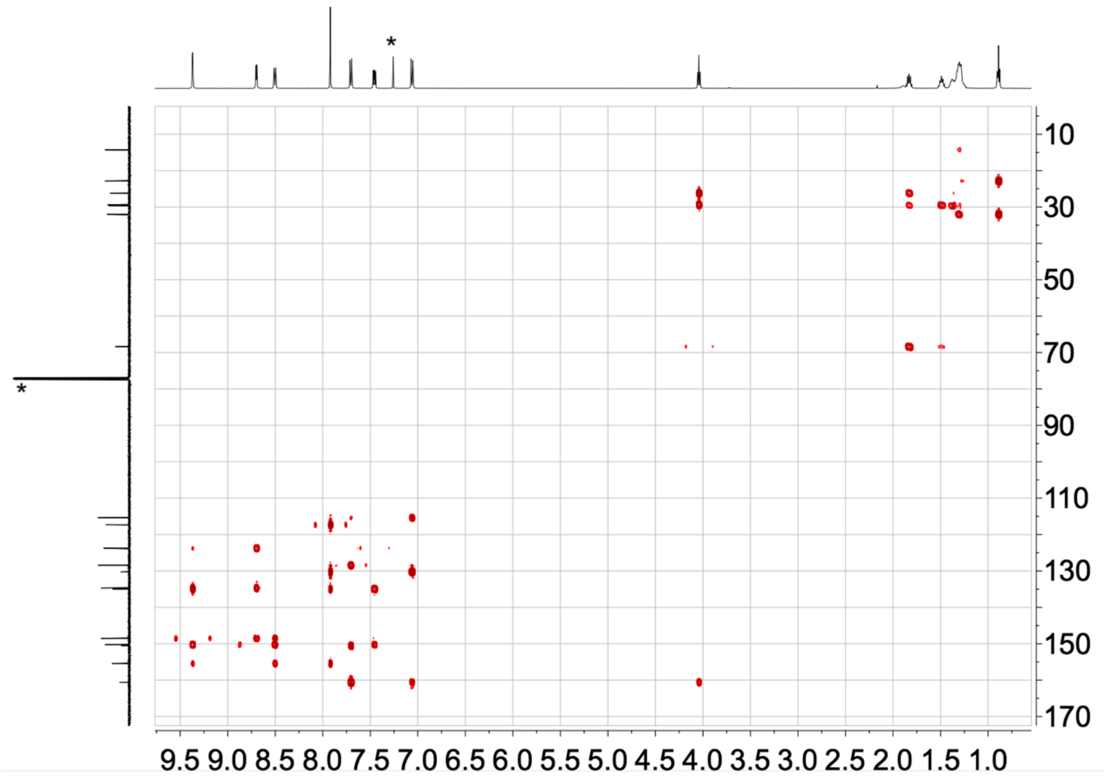

Figure S10. HMBC spectrum of compound **9** (^{1}H 500 MHz, ^{13}C 126 MHz, $CDCl_3$, 298 K). Scale: δ / ppm. * = residual $CHCl_3$ or $CDCl_3$.

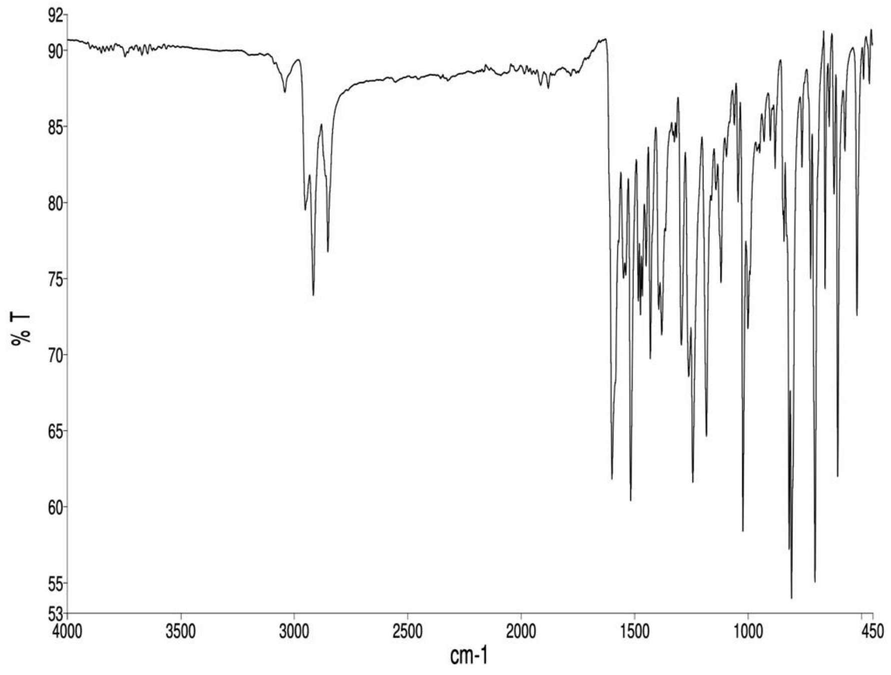

Figure 11. Solid-state FT-IR spectrum of compound **8**.

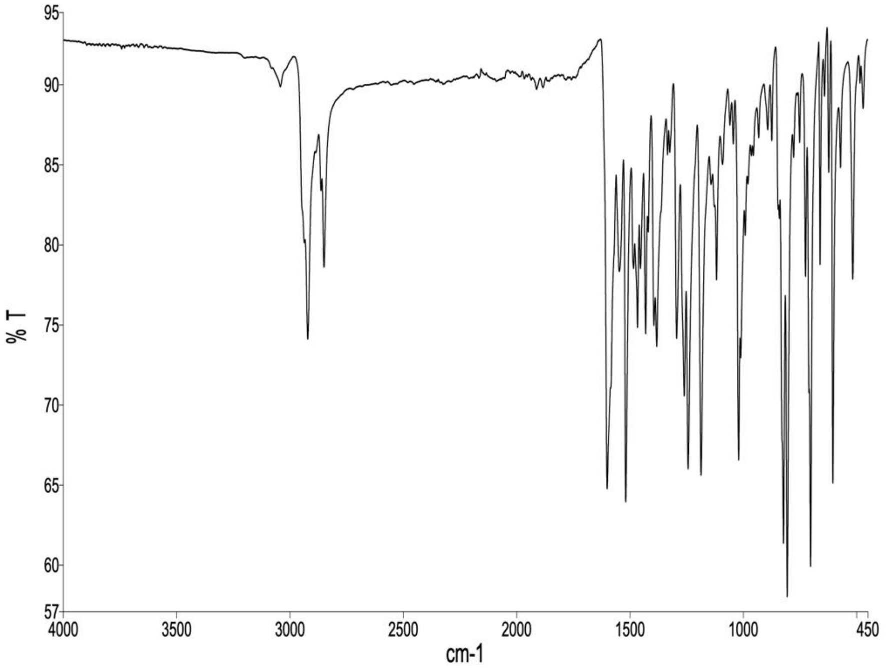

Figure 12. Solid-state FT-IR spectrum of compound **9**.

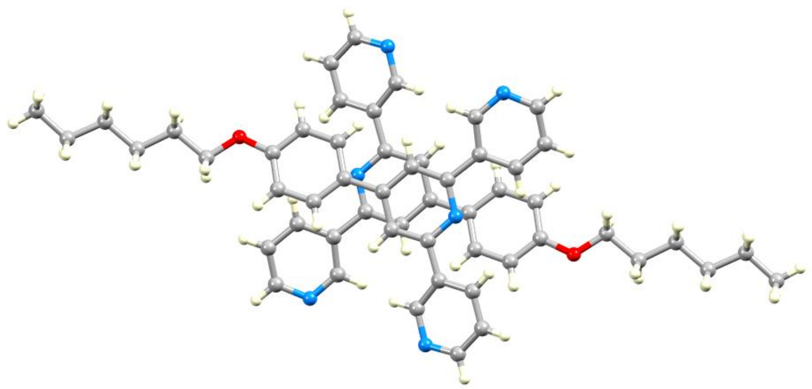

Figure S13. Head-to-tail (centrosymmetric) packing of 3,2':6',3"-tpy units in **6**.

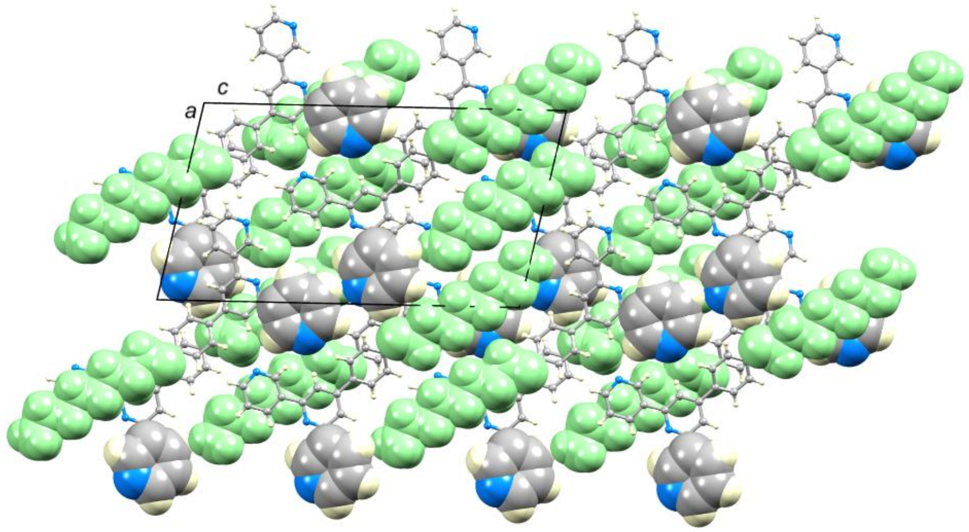

Figure S14. Part of two layers in the crystal lattice of **8** in which C–H$_{methylene}$... π interactions contribite to the packing.

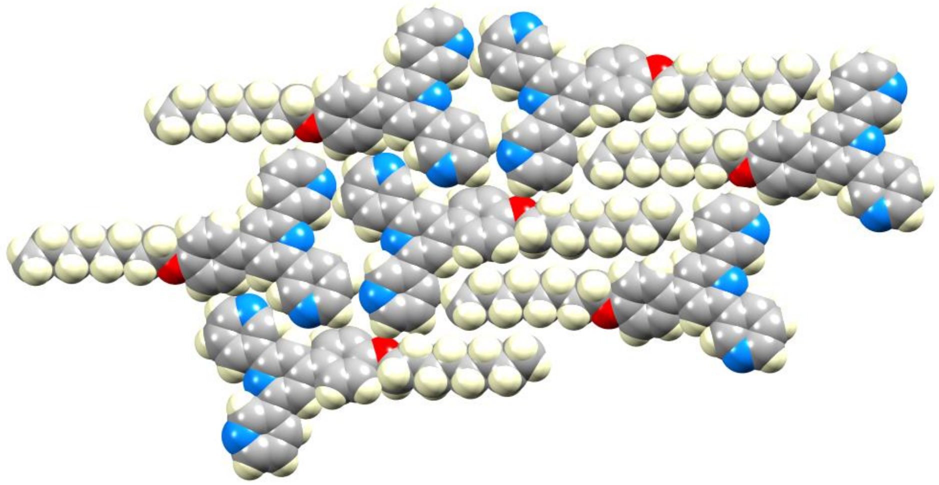

Figure S15. Space-filling representation of the packing diagram shown in the manuscript in Figure 8.

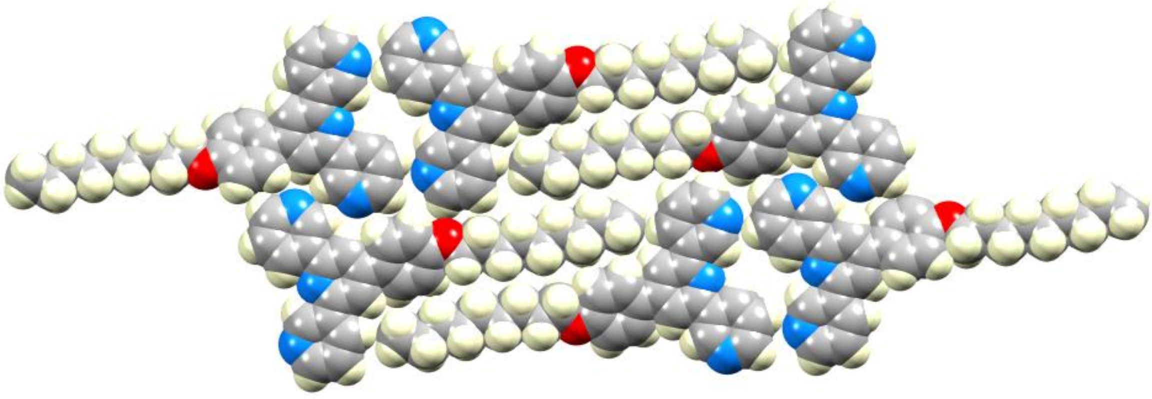

Figure S16. Space-filling representation of the packing diagram shown in the manuscript in Figure 10.
